# Supplementary material for: The androgen receptor is a therapeutic target in desmoplastic small round cell sarcoma
Source: Nat Commun. 2022 Jun 1;13:3057. doi: 10.1038/s41467-022-30710-z (PMC9160255; doi:10.1038/s41467-022-30710-z)
Supplement: Supplementary file 1 — Supplementary Information [file 41467_2022_30710_MOESM1_ESM.pdf]

Inventory of Supporting Information

• Antibodies used • Supplementary Figures and Legend • Supplementary Tables and Legend

Antibodies used for Western blots

Antibodies	Company
Androgen Receptor (D6F11) XP® Rabbit mAb #5153 (1:2000), Syk Antibody #2712 (1:1000), Tau (Tau46) Mouse mAb #4019 (1:1000), c-Kit Rabbit mAb #3074 (1:1000), Phospho-S6 Ribosomal Protein (Ser235/236) Antibody #2211 (1:1000), b-Actin mAb #3700 (1:1000), GAPDH (14C10) Rabbit mAb #2118 (1:1000), IGFBP2 Antibody #3922 (1:XX), Phospho-AMPKα (Thr172) (40H9) Rabbit mAb #2535 (1:1000), Akt (pan) (C67E7) Rabbit mAb #4691 (1:1000), Tuberin/TSC2 Antibody #3612 (1:1000), Integrin αV Antibody #4711 (1:1000), Integrin α4 Antibody #4600 (1:1000), Integrin α5 Antibody #4705 (1:1000), Integrin β5 Antibody #4708 (1:1000), Integrin β3 Antibody #4702 (1:1000), Integrin β1 Antibody #4706 (1:1000), SRC-1 (128E7) Rabbit mAb #2191 (1:1000), SRC-2 (D2X4M) Rabbit mAb #96687 (1:1000), SRC-3 (5E11) Rabbit mAb #2126 (1:1000).	Cell Signaling Technologies (CST)

Antibodies used for RPPA Analyses

#	Official Ab Name	Ab Name Reported on Dataset	Gene Name	Company
1	14-3-3 beta	14-3-3-beta	YWHAB	Santa Cruz
2	14-3-3 epsilon	14-3-3-epsilon	YWHAE	Santa Cruz
3	14-3-3 zeta	14-3-3-zeta	YWHAZ	Santa Cruz
4	4E-BP1	4E-BP1	EIF4EBP1	CST
5	4E-BP1_pS65	4E-BP1_pS65	EIF4EBP1	CST
6	4E-BP1_pT37_T46	4E-BP1_pT37_T46	EIF4EBP1	CST
7	53BP1	53BP1	TP53BP1	CST
8	Acetyl CoA Carboxylase 1	ACC1	ACACA	Epitomics
9	Acetyl-CoA Carboxylase_pS79	ACC_pS79	ACACA ACACB	CST
10	ACVRL1	ACVRL1	ACVRL1	Abcam
11	ADAR1	ADAR1	ADAR	Abcam
12	Akt	Akt	AKT1 AKT2 AKT3	CST
13	Akt_pS473	Akt_pS473	AKT1 AKT2 AKT3	CST
14	Akt_pT308	Akt_pT308	AKT1 AKT2 AKT3	CST
15	AMPKa	AMPK-alpha	PRKAA1	CST
16	AMPKa_pT172	AMPK-alpha_pT172	PRKAA1	CST
17	Androgen Receptor	AR	AR	Epitomics
18	Annexin I	Annexin-I	ANXA1	BD Biosciences
19	Annexin VII	Annexin-VII	ANXA7	BD Biosciences
20	A-Raf	A-Raf	ARAF	CST

21	ARHI	ARHI	DIRAS3	MDACC Laboratory
22	ATM	ATM	ATM	CST
23	ATM_pS1981	ATM_pS1981	ATM	CST
24	ATP5H	ATP5H	ATP5H	Abcam
25	ATR	ATR	ATR	CST
27	Bak	Bak	BAK1	Epitomics
28	BAP1	BAP1	BAP1	Santa Cruz
29	Bax	Bax	BAX	CST
30	BCL2	Bcl2	BCL2	Dako
31	Bcl-xL	Bcl-xL	BCL2L1	CST
32	Beclin	Beclin	BECN1	Santa Cruz
**#**	**Official Ab Name**	**Ab Name Reported on Dataset**	**Gene Name**	**Company**
33	beta-Catenin	b-Catenin	CTNNB1	CST
34	beta-Catenin_pT41_S45	b-Catenin_pT41_S45	CTNNB1	CST
35	Bid	Bid	BID	Abcam
36	Bim	Bim	BCL2L11	Abcam
37	B-Raf	B-Raf	BRAF	Santa Cruz
38	B-Raf_pS445	B-Raf_pS445	BRAF	CST
39	BRCA2	BRCA2	BRCA2	CST
40	Caspase-7 cleavedD198	Caspase-7-cleaved	CASP7	CST
41	Caspase-8	Caspase-8	CASP8	CST
42	Caveolin-1	Caveolin-1	CAV1	CST
43	CD29	CD29	ITGB1	BD Biosciences
44	CD31	CD31	PECAM1	Dako
45	CD49b	CD49b	ITGA2	BD Biosciences
46	cdc2/CDK1	CDK1	CDC2-CDK1	CST
47	CDKN2A/p16INK4a	p16INK4a	CDKN2A	Abcam
48	Chk1	Chk1	CHEK1	CST
49	Chk1_pS345	Chk1_pS345	CHEK1	CST
50	Chk2	Chk2	CHEK2	CST
51	Chk2_pT68	Chk2_pT68	CHEK2	CST
52	c-Jun_pS73	c-Jun_pS73	JUN	CST
53	c-Kit	c-Kit	KIT	Abcam
54	Claudin 7	Claudin-7	CLDN7	Novus Biologicals
55	c-Met	c-Met	MET	CST
56	c-Met_pY1234_Y1235	c-Met_pY1234_Y1235	MET	CST
57	c-Myc	c-Myc	MYC	Santa Cruz
58	COL6A1	Collagen-VI	COL6A1	Santa Cruz
59	Complex II Subunit	Complex-II-Subunit	SDHA	Invitrogen
60	Cox IV	Cox-IV	COX4I1	Abcam
61	Cox2	Cox2	PTGS2	CST
62	C-Raf/Raf-1	C-Raf	RAF1	Millipore
63	C-Raf_pS338	C-Raf_pS338	RAF1	CST
64	Cyclin B1	Cyclin-B1	CCNB1	Epitomics
65	Cyclin D1	Cyclin-D1	CCND1	Santa Cruz
66	Cyclin E1	Cyclin-E1	CCNE1	Santa Cruz
67	Cyclophilin F	Cyclophilin-F	PPIF	Abcam

68	Dvl3	Dvl3	DVL3	CST
69	E2F-1	E2F1	E2F1	Santa Cruz
70	E-Cadherin	E-Cadherin	CDH1	CST
71	eEF2	eEF2	EEF2	CST
72	eEF2K	eEF2K	EEF2K	CST
73	EGFR	EGFR	EGFR	CST
74	EGFR_pY1068	EGFR_pY1068	EGFR	CST
75	EGFR_pY1173	EGFR_pY1173	EGFR	Abcam
76	eIF4E	eIF4E	EIF4E	CST
77	eIF4G	eIF4G	EIF4G1	CST
78	ER alpha_pS118	ER-alpha_pS118	ESR1	Epitomics
79	ErbB2/HER2	HER2	ERBB2	Lab Vision
**#**	**Official Ab Name**	**Ab Name Reported on Dataset**	**Gene Name**	**Company**
80	ErbB2/HER2_pY1248	HER2_pY1248	ERBB2	R&D Systems
81	ErbB3/HER3	HER3	ERBB3	Santa Cruz
82	ErbB3/HER3_pY1289	HER3_pY1289	ERBB3	CST
83	ERCC1	ERCC1	ERCC1	Santa Cruz
84	ERRFI1/MIG6	MIG6	ERRFI1	Sigma-Aldrich
85	Estrogen Receptor	ER-alpha	ESR1	Lab Vision
86	Ets-1	Ets-1	ETS1	Bethyl
87	FAK	FAK	PTK2	CST
88	FAK_pY397	FAK_pY397	PTK2	CST
89	Fatty Acid Synthase	FASN	FASN	CST
90	Fibronectin	Fibronectin	FN1	Epitomics
91	FoxM1	FoxM1	FOXM1	CST
92	FoxO3a	FoxO3a	FOXO3	CST
93	FoxO3a_pS318_S321	FoxO3a_pS318_S321	FOXO3	CST
94	G6PD	G6PD	G6PD	Santa Cruz
95	Gab2	Gab2	GAB2	CST
96	GAPDH	GAPDH	GAPDH	Life Technologies
97	GATA3	GATA3	GATA3	BD Biosciences
98	GCN5L2	GCN5L2	KAT2A	CST
99	Glycogen Synthase	Gys	GYS1	CST
100	Glycogen Synthase_pS641	Gys_pS641	GYS1	CST
101	GPBB	GPBB	PYGB	Novus Biologicals
102	GSK-3alpha/beta	GSK-3ab	GSK3A<br>GSK3B	Santa Cruz
103	GSK-3alpha/beta_pS21_S9	GSK-3ab_pS21_S9	GSK3A<br>GSK3B	CST
104	GSK-3beta_pS9	GSK-3b_pS9	GSK3B	CST
105	Heregulin	Heregulin	NRG1	CST
106	HIAP	HIAP	BIRC2	Millipore
107	Histone H3	Histone-H3	H3F3A<br>H3F3B	Abcam
108	IGF-1Receptor beta	IGF1R-beta	IGF1R	CST
109	IGFBP2	IGFBP2	IGFBP2	CST
110	INPP4b	INPP4b	INPP4B	CST
111	IRS1	IRS1	IRS1	Millipore

112	JAB1	JAB1	COPS5	Santa Cruz
113	JNK/SAPK_pT183_Y185	JNK_pT183_Y185	MAPK8	CST
114	JNK2	JNK2	MAPK9	CST
115	KMT3A/HYPB/HIF 1	SETD2	SETD2	Abcam
116	Lck	Lck	LCK	CST
117	MAPK_pT202_Y204	MAPK_pT202_Y204	MAPK1<br>MAPK3	CST
118	Mcl 1	Mcl-1	MCL1	CST
119	MDM2_pS166	MDM2_pS166	MDM2	CST
120	MEK1	MEK1	MAP2K1	Epitomics
121	MEK1_pS217_S221	MEK1_pS217_S221	MAP2K1<br>MAP2K2	CST

#	Official Ab Name	Ab Name Reported on Dataset	Gene Name	Company
122	MEK2	MEK2	MAP2K2	CST
123	Merlin/NF2	Merlin	NF2	Novus Biologicals
124	MSH2	MSH2	MSH2	CST
125	MSH6	MSH6	MSH6	Novus Biologicals
126	mTOR	mTOR	MTOR	CST
127	mTOR_pS2448	mTOR_pS2448	MTOR	CST
128	Myosin heavy chain 11	Myosin-11	MYH11	Novus Biologicals
129	Myosin IIa_pS1943	Myosin-IIa_pS1943	MYH9	CST
130	NAPSIN A	NAPSIN-A	NAPSA	Abcam
131	N-Cadherin	N-Cadherin	CDH2	CST
132	NDRG1_pT346	NDRG1_pT346	NDRG1	CST
133	NF-kappaB p65_pS536	NF-kB-p65_pS536	RELA	CST
134	Notch1	Notch1	NOTCH1	CST
135	N-Ras	N-Ras	NRAS	Santa Cruz
136	p21	p21	CDKN1A	Santa Cruz
137	p27 KIP 1	p27-Kip-1	CDKN1B	Abcam
138	p27 KIP 1_pT198	p27_pT198	CDKN1B	Abcam
139	p27/Kip1_pT157	p27_pT157	CDKN1B	R&D Systems
140	p38 alpha MAPK	p38-alpha	MAPK14	CST
141	p38 MAPK	p38	MAPK14	CST
142	p38 MAPK_pT180_Y182	p38_pT180_Y182	MAPK14	CST
143	p53	p53	TP53	CST
144	p70 S6 Kinase_pT389	p70-S6K_pT389	RPS6KB1	CST
145	p70/S6K1	p70-S6K1	RPS6KB1	Epitomics
146	PAI-1	PAI-1	SERPINE1	BD Biosciences
147	PARK7/DJ1	DJ1	PARK7	Abcam
148	PARP cleavedD214	PARP-cleaved	PARP1	CST
149	PARP-1	PARP1	PARP1	Santa Cruz
150	Paxillin	Paxillin	PXN	Epitomics
151	PCNA	PCNA	PCNA	Abcam
152	Pdcd-1L1	Pdcd-1L1	CD274	Santa Cruz
153	Pdcd4	Pdcd4	PDCD4	Rockland
154	PDGFR beta	PDGFR-beta	PDGFRB	CST
155	PDK1	PDK1	PDPK1	CST
156	PDK1_pS241	PDK1_pS241	PDPK1	CST

157	PEA-15	PEA-15	PEA15	CST
158	PED/PEA-15_pS116	PEA-15_pS116	PEA15	Invitrogen
159	PI3 Kinase p110 alpha	PI3K-p110-alpha	PIK3CA	CST
160	PI3K p85	PI3K-p85	PIK3R1	Millipore
161	PKC alpha	PKC-alpha	PRKCA	Millipore
162	PKC alpha_pS657	PKC-alpha_pS657	PRKCA	Millipore
163	PKC beta II_pS660	PKC-beta-II_pS660	PRKCA<br>PRKCB<br>PRKCD<br>PRKCE<br>PRKCH<br>PRKCQ	CST

#	Official Ab Name	Ab Name Reported on Dataset	Gene Name	Company
164	PKC delta_pS664	PKC-delta_pS664	PRKCD	Millipore
165	PMS2	PMS2	PMS2	Novus Biologicals
166	PRAS40_pT246	PRAS40_pT246	AKT1S1	Life Technologies
167	PREX1	PREX1	PREX1	Abcam
168	Progesterone Receptor	PR	PGR	Abcam
169	PTEN	PTEN	PTEN	CST
170	Rab11	Rab11	RAB11A<br>RAB11B	CST
171	Rab25	Rab25	RAB25	CST
172	Rad50	Rad50	RAD50	Millipore
173	Rad51	Rad51	RAD51	CST
174	Raptor	Raptor	RPTOR	CST
175	Rb	Rb	RB1	CST
176	Rb_pS807_S811	Rb_pS807_S811	RB1	CST
177	RBM15	RBM15	RBM15	Novus Biologicals
178	Rictor	Rictor	RICTOR	CST
179	Rictor_pT1135	Rictor_pT1135	RICTOR	CST
180	RSK	RSK	RPS6KA1<br>RPS6KA2<br>RPS6KA3	CST
181	S6_pS235_S236	S6_pS235_S236	RPS6	CST
182	S6_pS240_S244	S6_pS240_S244	RPS6	CST
183	SCD	SCD	SCD	Santa Cruz
184	SF2/ASF	SF2	SRSF1	Invitrogen
185	Shc_pY317	Shc_pY317	SHC1	CST
186	Smac/Diablo	Smac	DIABLO	CST
187	Smad1	Smad1	SMAD1	Epitomics
188	Smad3	Smad3	SMAD3	Abcam
189	Smad4	Smad4	SMAD4	Santa Cruz
190	Snail	Snail	SNAI1	CST
191	Src	Src	SRC	Millipore
192	Src Family_pY416	Src_pY416	SRC<br>LYN<br>FYN<br>LCK	CST

			YES1<br>HCK	
193	Src_pY527	Src_pY527	SRC<br>YES1<br>FYN<br>FGR	CST
194	Stat3_pY705	Stat3_pY705	STAT3	CST
195	Stat5a	Stat5a	STAT5A	Abcam
196	Stathmin 1	Stathmin-1	STMN1	Abcam
197	Syk	Syk	SYK	Santa Cruz
198	TAZ	TAZ	WWTR1	CST
199	TIGAR	TIGAR	C12ORF5	Abcam
200	Transferrin Receptor	TFRC	TFRC	Novus Biologicals
**#**	**Official Ab Name**	**Ab Name Reported on Dataset**	**Gene Name**	**Company**
201	Transglutaminase II	Transglutaminase	TGM2	Lab Vision
202	TSC1/Hamartin	TSC1	TSC1	CST
203	TSC2 Tuberin_pT1462	Tuberin_pT1462	TSC2	CST
204	TTF1	TTF1	NKX2-1	Abcam
205	Tuberin	Tuberin	TSC2	Epitomics
206	Twist	TWIST	TWIST2	Santa Cruz
207	Tyro3	Tyro3	TYRO3	CST
208	UBAC1	UBAC1	UBAC1	Sigma-Aldrich
209	UGT1A	UGT1A	UGT1A1	Santa Cruz
210	UQCRC2	UQCRC2	UQCRC2	Abcam
211	VDAC1/Porin	Porin	VDAC1	Abcam
212	VEGF Receptor 2	VEGFR-2	KDR	CST
213	VHL	VHL	VHL	BD Biosciences
214	XRCC1	XRCC1	XRCC1	CST
215	YAP	YAP	YAP1	Santa Cruz
216	YAP_pS127	YAP_pS127	YAP1	CST
217	YB1	YB1	YBX1	Novus Biologicals
218	YB1_pS102	YB1_pS102	YBX1	CST

SUPPLEMENTAL TABLE

Supplemental Table 1. Demographic information for DSRCT and Ewing sarcoma patients in RPPA analysis.

Sample ID	Age range at diagnosis	Gender	PDX-Development	Final Diagnosis	Tumor Status	Chemo pre-treatment
**DSRCT-1T**	25-29	Male	NA	DSRCT	primary	yes
**DSRCT-T2**	20-24	Male	NA	DSRCT	primary	yes
**DSRCT-T3**	9-10	Male	NA	DSRCT	progression of residual tumor	yes
**DSRCT-T4**	45-49	Male	NA	DSRCT	primary	yes
**DSRCT-T5**	20-24	Male	NA	DSRCT	primary	yes
**DSRCT-T6**	50-54	Male	NA	DSRCT	primary	yes
**DSRCT-T7**	10-14	Female	NA	DSRCT	recurrent	yes
**DSRCT-T8**	45-49	Male	NA	DSRCT	recurrent	yes
**DSRCT-T9**	25-29	Male	NA	DSRCT	primary	yes
**DSRCT-T10**	15-19	Male	NA	DSRCT	primary	yes
**DSRCT-T11**	15-19	Male	NA	DSRCT	primary	yes
**DSRCT-T12**	15-19	Male	NA	DSRCT	primary	yes
**DSRCT-T13**	15-19	Male	NA	DSRCT	primary	yes
**DSRCT-T14**	15-19	Male	NA	DSRCT	primary	yes
**DSRCT-T15**	9-10	Male	PDX1	DSRCT	primary	yes
**DSRCT-T16**	40-44	Male	NA	DSRCT	primary	yes
**ES1**	15-19	Male	NA	ES	primary	yes
**ES2**	30-34	Male	NA	ES	primary	yes
**ES3**	25-29	Female	NA	ES	primary	yes
**ES4**	20-24	Male	NA	ES	primary	yes
**ES5**	45-49	Female	NA	ES	primary	yes
**ES6**	20-24	Male	NA	ES	primary	yes

SUPPLEMENTAL FIGURES

Supplemental Figure 1

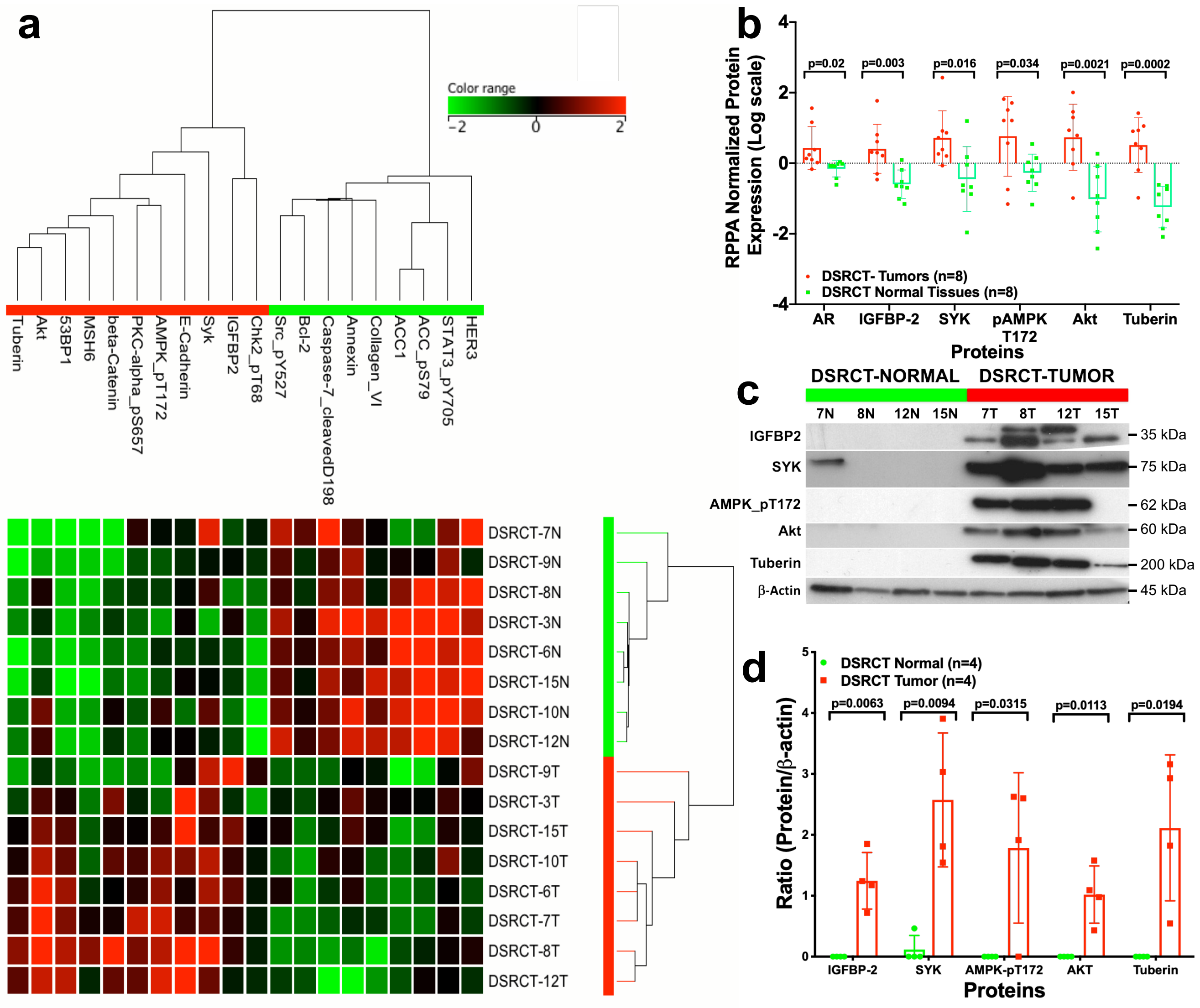

Supplemental Figure 1. Proteomic signature differentiates DSRCT from normal mesenteric tissues. (**a**) Proteomic profiling between eight DSRCT and eight normal mesenteric tissue specimens. The protein lysates from DSRCT (red) and normal mesenteric tissue (green) were subjected to RPPA analysis for 151 proteins and phosphoproteins (red, increased signal; green, decreased signal). Unsupervised double-hierarchical clustering using the Pearson correlation distance metric between proteins (rows) and Centroid linkage (a clustering method) separated the 16 samples into two groups (columns). The heat map of all 16 samples showed 20 proteins that distinguished DSRCT from normal mesenteric tissues. Two-sided T-test with p ≤0.05; fold-change ≥2). (**b**) Normalized RPPA protein expression relative to β-actin. P value was calculated by two-sided T-test (**c**) Western blotting of four matching samples was used to validate the proteins identified by RPPA as being differentially expressed between DSRCT and normal mesenteric tissue. (**d**) Normalized protein expression relative to β-actin. *P* value was calculated by two-sided T-test. Bar graphs in **b** and **d** are presented as mean value ± SD. n=number of tumor or normal samples analyzed.

Supplemental Figure 2

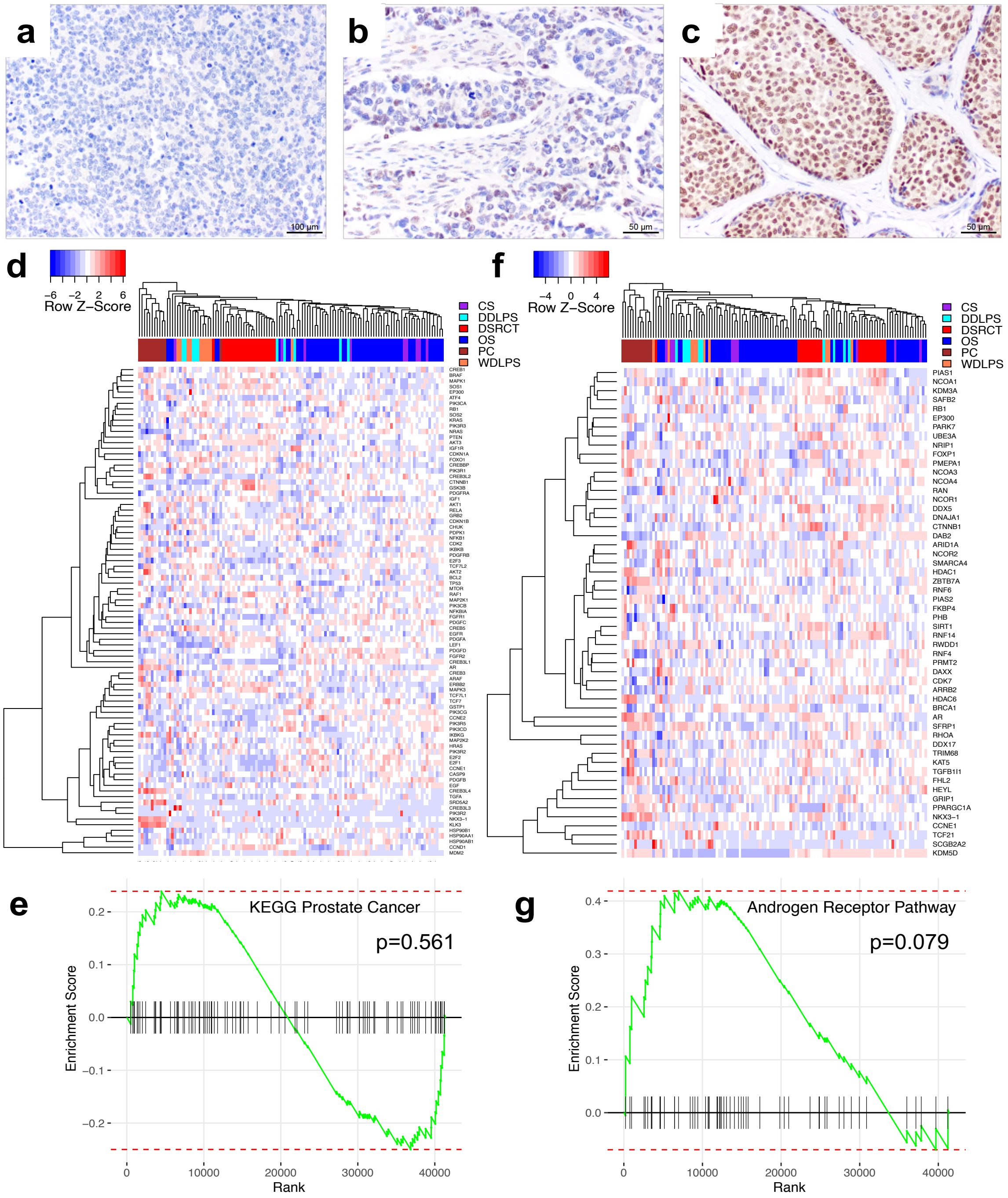

Supplemental Figure 2. Proteomic and genomic profiling of DSRCT and other types of sarcoma primary tumor samples. (a-c) Examples of TMA immunohistochemical labeling in desmoplastic small round cell tumor. (**a**) Complete absence of tumoral nuclear labeling for AR (100x). (**b)** Variable tumoral labeling for AR (200x), with a mixture of tumor cells positive (brown nuclei) and absent (blue nuclei) for nuclear labeling. The extent of labeling was estimated as the percentage of all labeled cells. **(c)** Diffuse nuclear tumoral labeling for AR (200x). **(d)** Heatmap of the KEGG Prostate Cancer pathway and (**e**) the corresponding GSEA plot. (**f)** Heatmap of the GO Androgen Receptor Signaling Pathway and (**g)** the corresponding GSEA plot. The Z-score normalized expression levels of the genes that make up the KEGG Prostate Cancer and GO Androgen Receptor Signaling pathways were used in unsupervised clustering to generate the heatmaps. DSRCT (n=22); PC=prostate cancer (n=12); OS=osteosarcoma (n=47); CS=chondrosarcoma (n=7); WDLPS=well-differentiated liposarcoma (n=7), and DDLPS=dedifferentiated liposarcoma (n=10).

Supplemental Figure 3

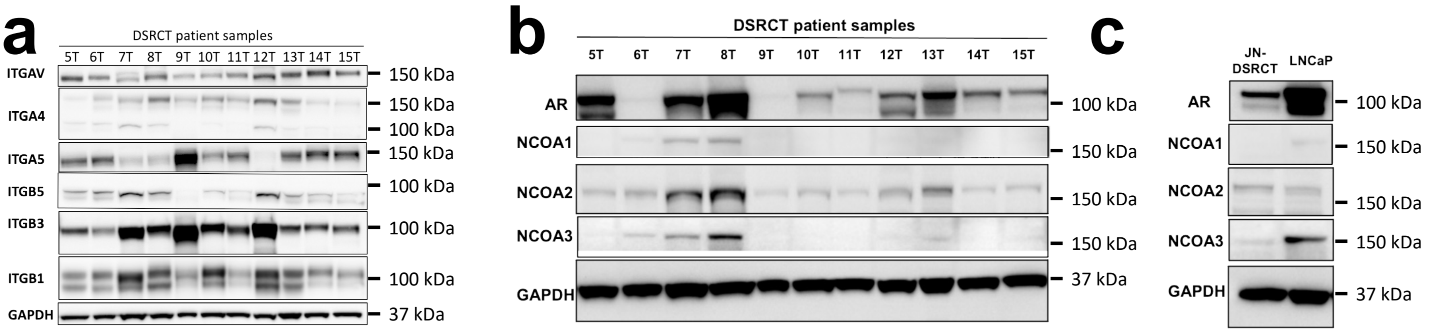

Supplemental Figure 3. Additional proteomic profiling of DSRCT samples using western blotting analyses. (a) Western blot analyses of integrin subclasses (ITGAV, ITGA4, ITGA5, ITGB1, ITGB3, and ITGB5) and GAPDH expression in 11 DSRCT cohort primary tumors. **(b)** Western blot analyses of steroid receptor coactivators subclasses (NCOA1: SRC-1, NCOA2: SRC-2, and NCOA3: SRC-3) and GAPDH expression in 11 DSRCT cohort primary tumors. **(c)** Western blot analyses of steroid receptor coactivators subclasses (NCOA1: SRC-1, NCOA2: SRC-2, and NCOA3: SRC-3) and GAPDH expression in JN-DSRCT and LNCaP PC cell lines.

Supplemental Figure 4

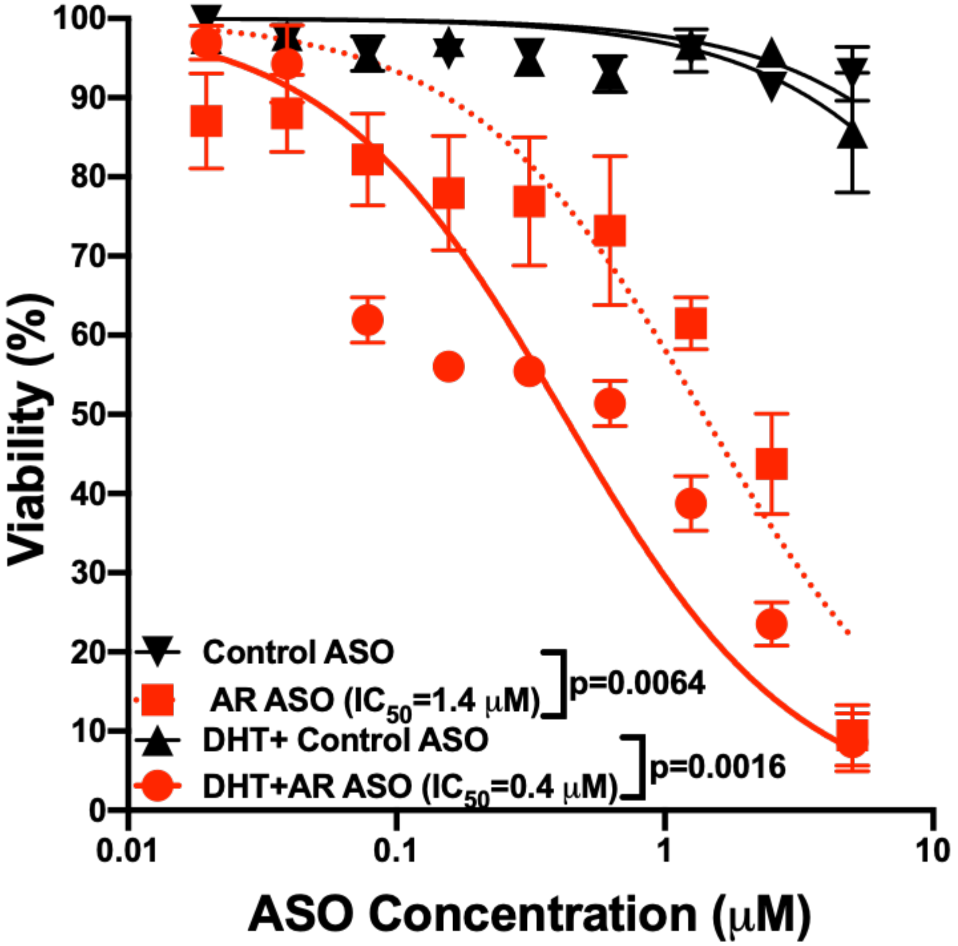

Supplemental Figure 4. DHT treatment of JN-DSRCT cells enhanced their sensitivity towards AR-based antisense blockade. WST1 proliferation cell-based assay testing AR-ASO and control ASO sequences in a dose-dependent manner for 72 hours in the presence or the absence of DHT. Data points represent mean ± SEM using three experimental replicates for each cell line. *P* values calculated by unpaired two-tailed T-test.

Supplemental Figure 5

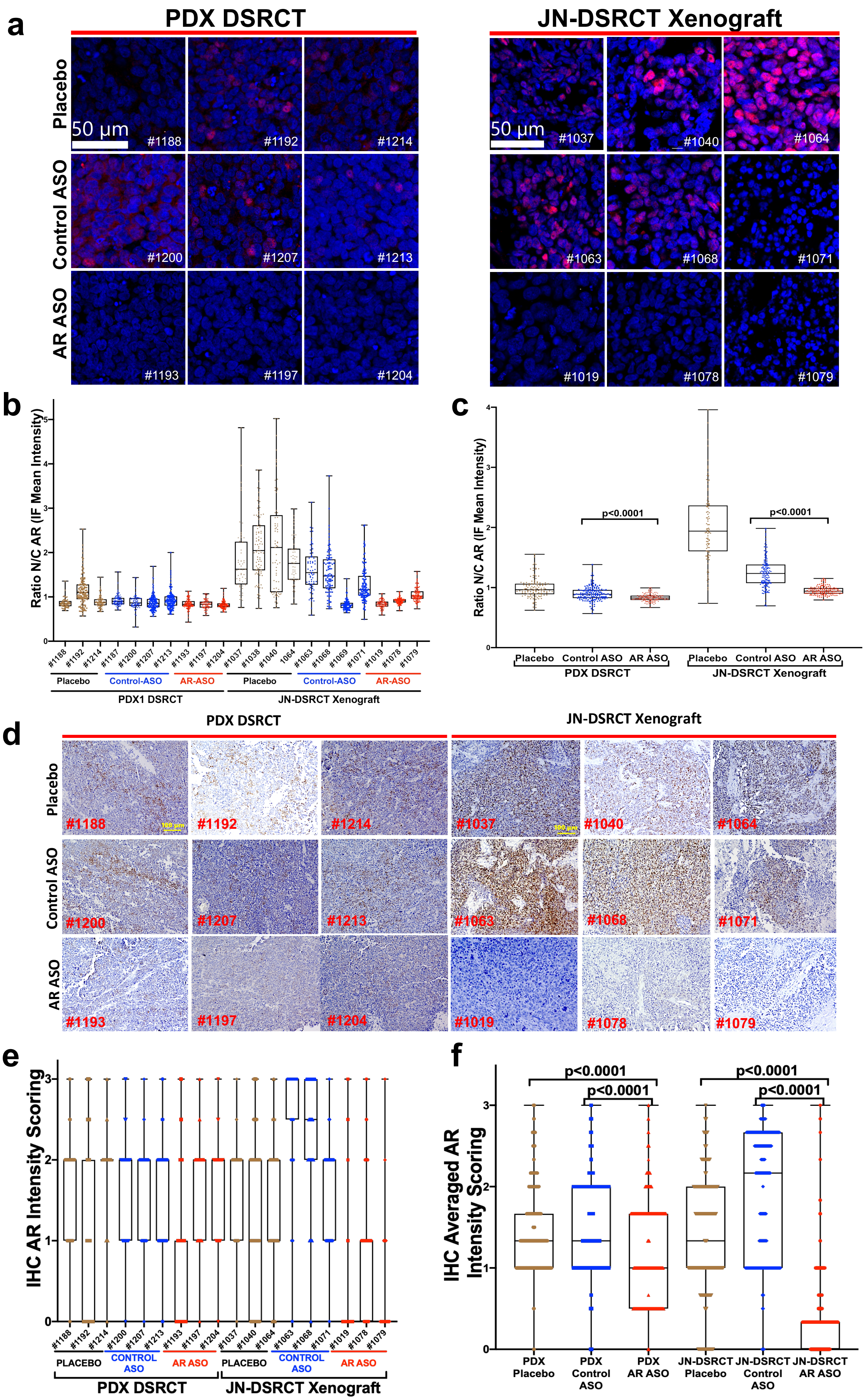

Supplemental Figure 5. Immunofluorescence confocal and IHC evaluation of AR expression in JN-DSRCT and PDX tumors after AR-based antisense therapy. **(a)** Immunofluorescence evaluation images of preclinical JN-DSRCT and PDX tumor samples. **(b)** Representative Nuclear/Cytoplasmic ratio AR immunofluorescence confocal microscopy quantification, within the single cell or, **(c)** the averaged treated samples (placebo, control ASO, and AR ASO). **(d)** Immunohistochemical evaluation images of preclinical JN-DSRCT and PDX tumor samples. IHC stains for AR in primary tumors of JN-DSRCT and PDX DSRCT mice after treatment with AR ASO, control ASO, and placebo. 100μm scale bars are shown. **(e)** Representative IHC AR intensity scoring of the preclinical JN-DSRCT and PDX tumor samples, within the single cell or, **(f)** the averaged treated samples (placebo, control ASO, and AR ASO). *P* value was calculated by two-sided T-test. Box plots in **b, c, e** and **f** are presented as median value ± SD, interquartile range, and minimum and maximum data points. *P* values calculated by unpaired two-tailed T-test.

Supplemental Figure 6

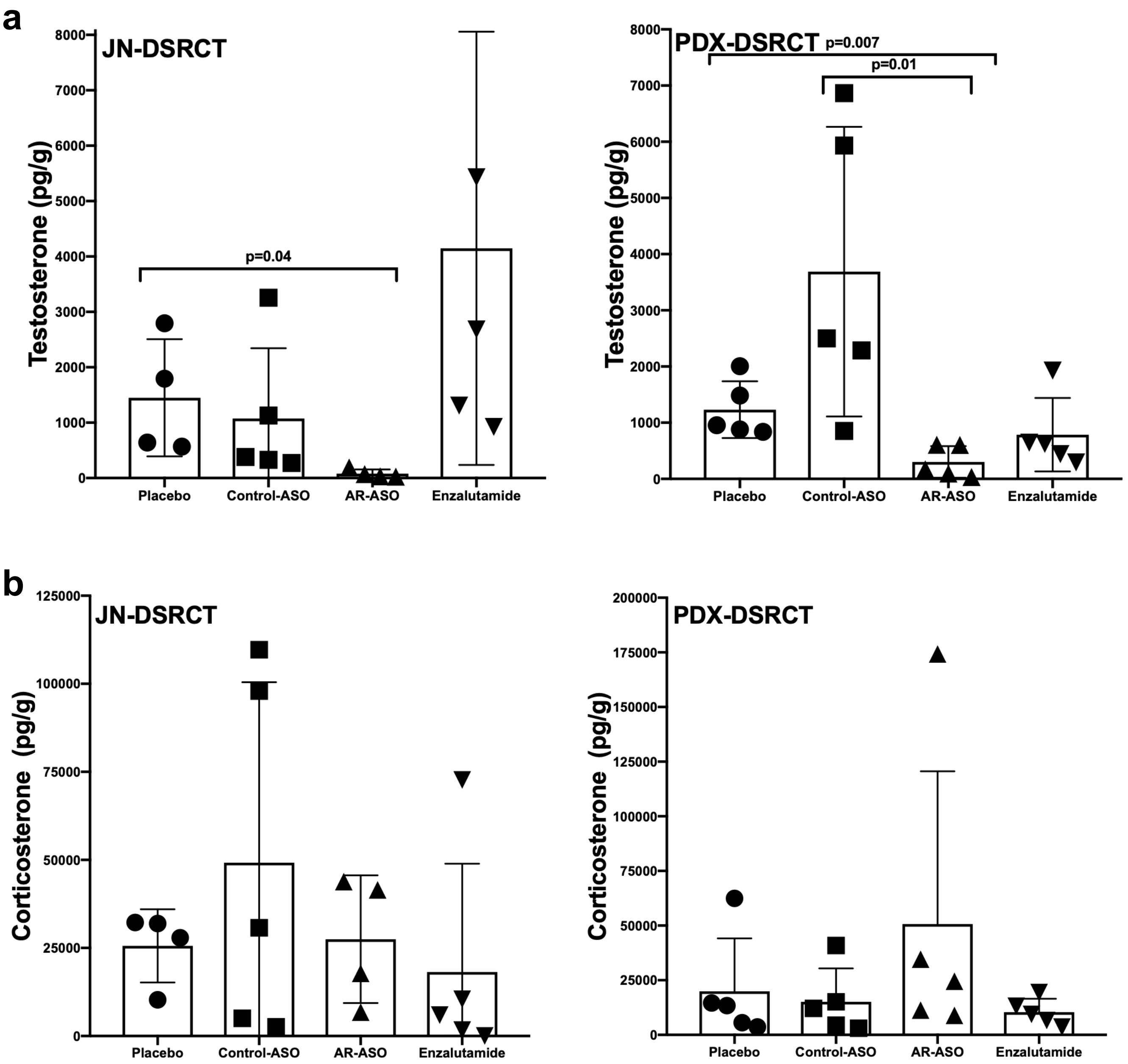

Supplemental Fig 6. Testosterone and corticosterone quantification in DSRCT preclinical animal samples. Testosterone (panel **a**) and corticosterone (panel **b**) levels (pg/g) in JN-DSRCT xenograft and patient-derived tumor explants (PDX-DSRCT). Testosterone levels were statistically dissimilar in JN-DSRCT and PDX-DSRCT placebo versus AR-ASO with *P* values of p=0.04 and p=0.007, respectively, using a two-sided T-test. In the PDX-DSRCT, the control-ASO and AR-ASO testosterone levels were statistically different (p=0.01). The corticosterone levels were similar across all treatments in both JN-DSRCT and PDX-DSRCT tumor models.

Supplemental Figure 7

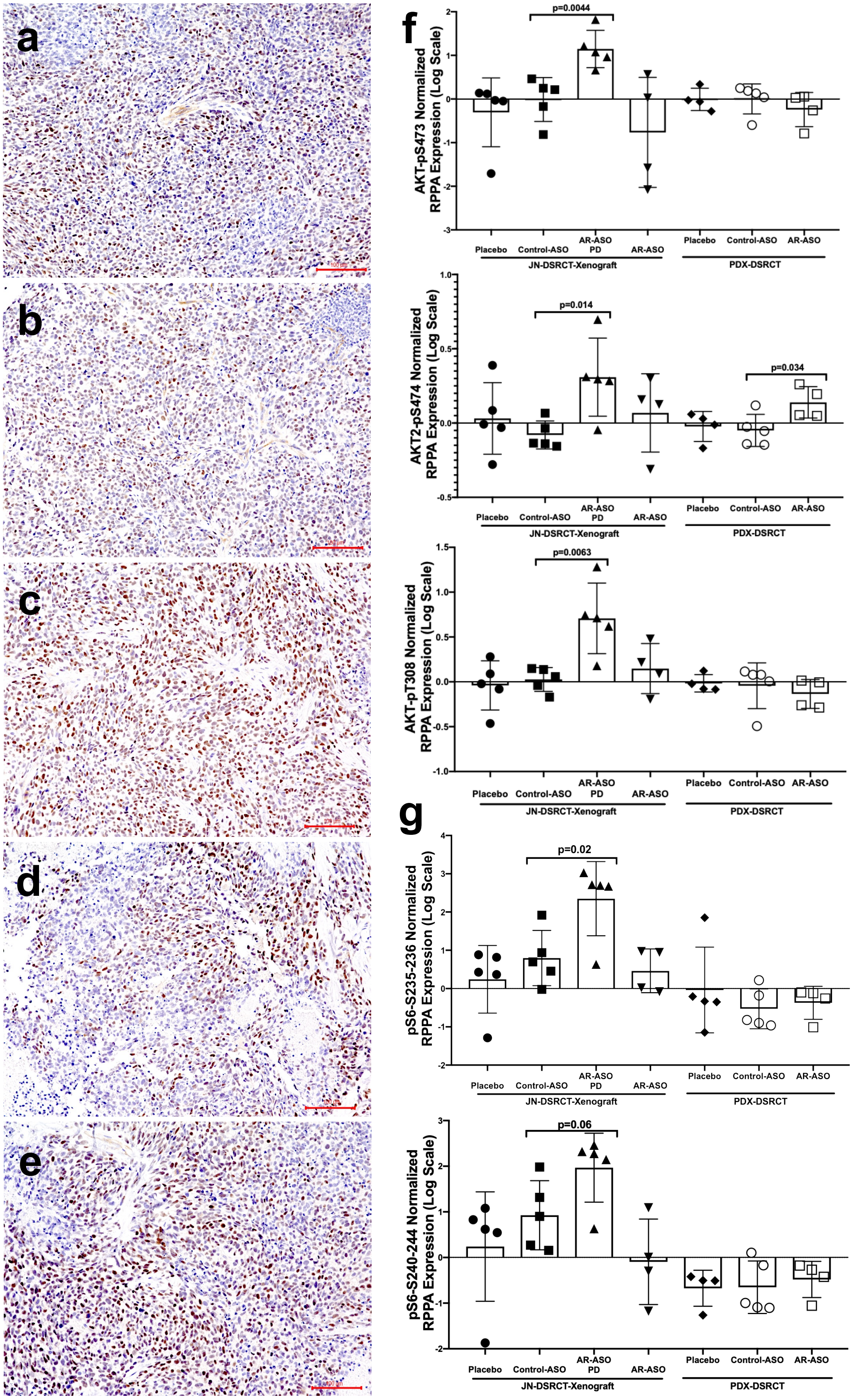

Supplemental Fig 7. Proteomic evaluation of AR expression in preclinical JN-DSRCT tumors after enzalutamide-based therapy. (a-e) IHC stains for AR in primary tumors of JN-DSRCT mice after treatment with enzalutamide. Representative images from five enzalutamide-treated JN-DSRCT xenograft mice are shown. 100μm scale bars are shown. **(f)** The mean expression intensity values of the three AKT-pS473, AKT2-pS474, and AKT-pT308 proteins associated with different group of mice treatments in JN-DSRCT xenograft and PDX animal models of DSRCT, and their statistical significance after normalization for global protein expression by median centering across 151 antibodies in the RPPA panel. **(g)** The mean expression intensity values of the two pS6-S235-236, and pS6-S240-244 proteins associated with different group of mice treatments in JN-DSRCT xenograft and PDX animal models of DSRCT, and their statistical significance after normalization for global protein expression by median centering across 151 antibodies in the RPPA panel. *P* values calculated by unpaired two-tailed T-test.

Supplemental Figure 8

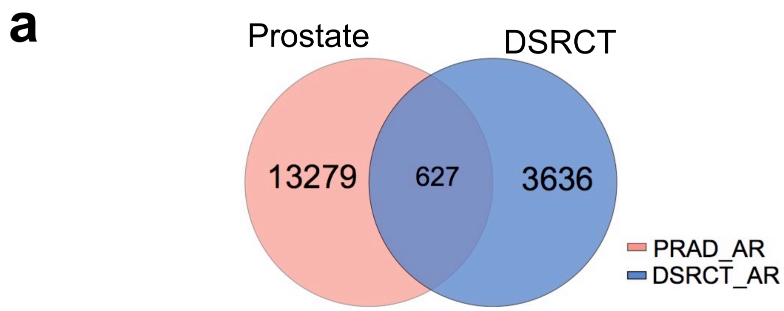

b

Top 5 AR common binding Motifs			
TF Class	TF		P-value
Nuclear receptor		GRE	1e-201
		ARE	1e-197
		PGR	1e-151
Fork-head		FOXM1	1e-166
		FOXA1	1e-156

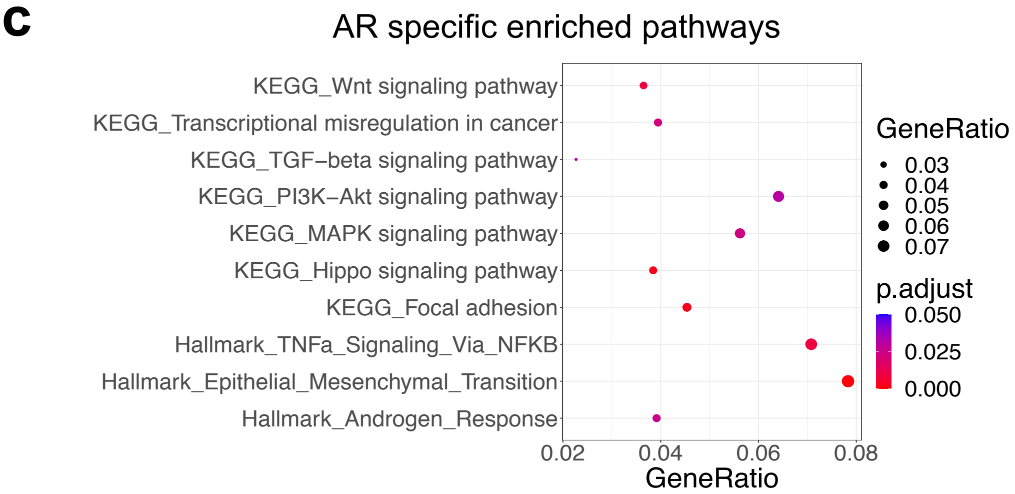

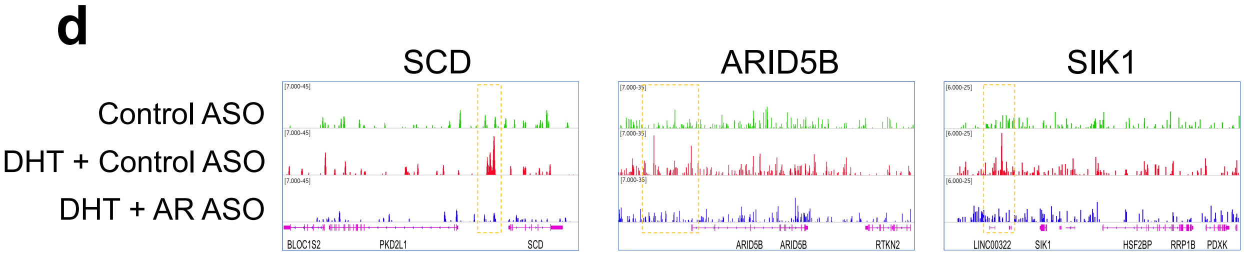

Supplemental Figure 8. AR binding in DSRCT cells. (a) Venn diagram showing the overlap of AR peaks between DHT + Control ASO DSRCT and PRAD 22RV1 samples to identify the shared AR binding sites. **(b)** List of top 5 enriched transcription factor (TF) motifs in DSRCT and PRAD shared AR binding sites. Motifs are identified using HOMER (Binomial test). **(c)** List of enriched pathways for genes that harbor AR binding sites in their vicinity in DHT-treated DSRCT cells. Dot plot showing significantly enriched pathways for AR specific binding sites. Dot size represents gene ratio, and colors represent adjusted *p*-values (Fisher's exact test). **(d)** IGV images showing enrichment of AR peaks around SCD, ARID5B, and SIK1 genes using aggregate ChIP-seq profiles of Control ASO, DHT + Control ASO, and DHT + AR ASO samples.

Supplemental Figure 9

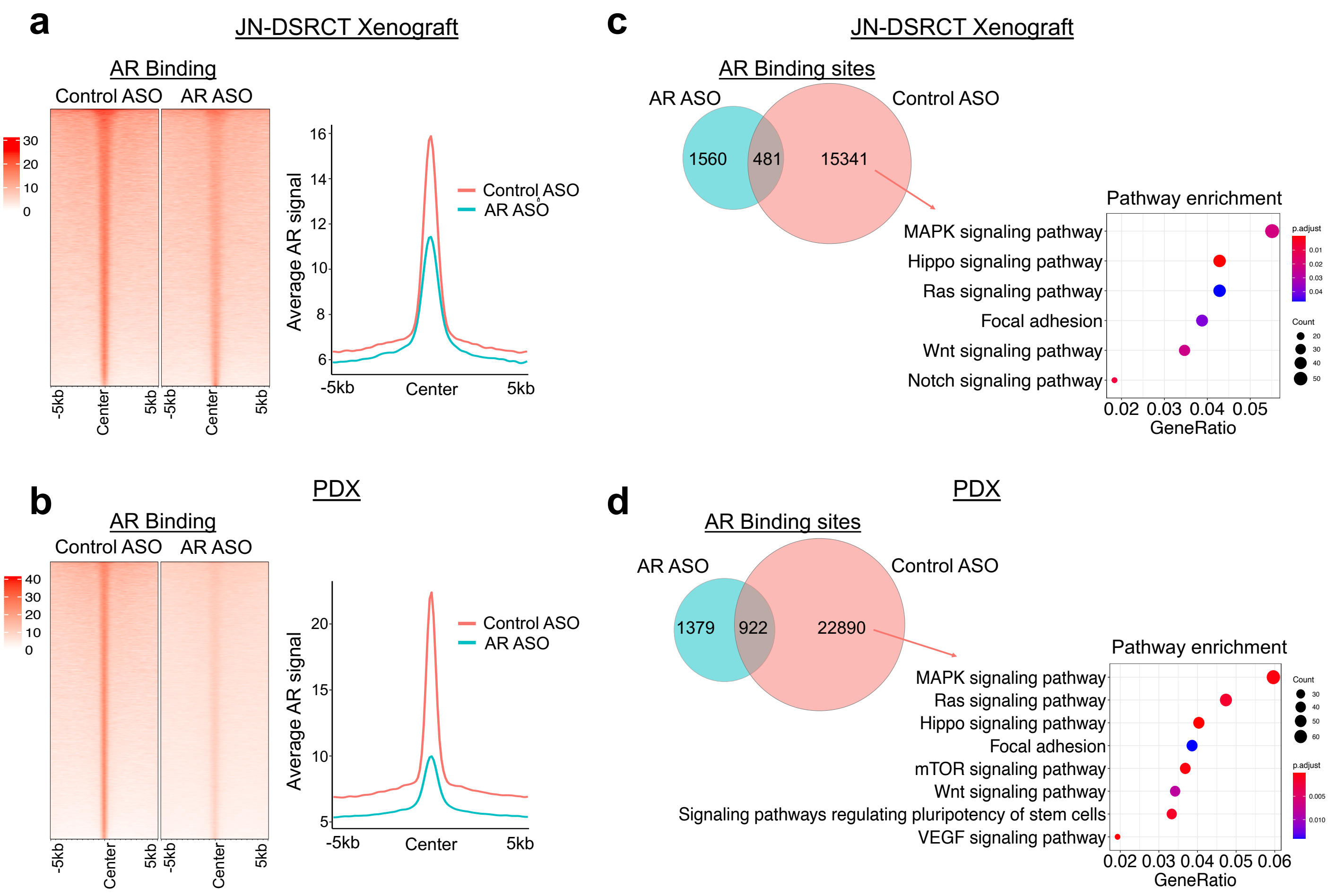

e

JN-DSRCT Xenograft<br>AR specific binding Motifs		PDX<br>AR specific binding Motifs	
TF	P-value		
AR-halfsite	1e-8	AR-halfsite	1e-7
OCT	1e-8	c-Myc	1e-4
c-Myc	1e-4	Foxo1	1e-2
Foxa2	1e-3	OCT	1e-2
p53	1e-3	ERG	1e-2

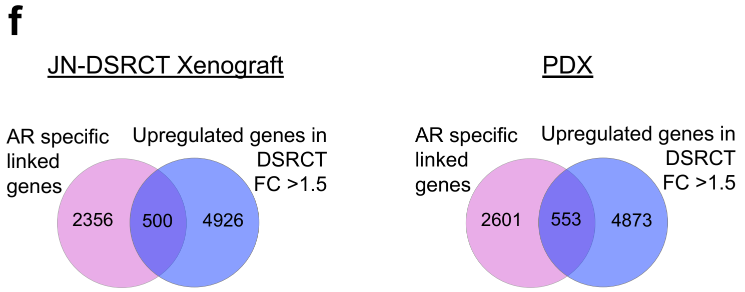

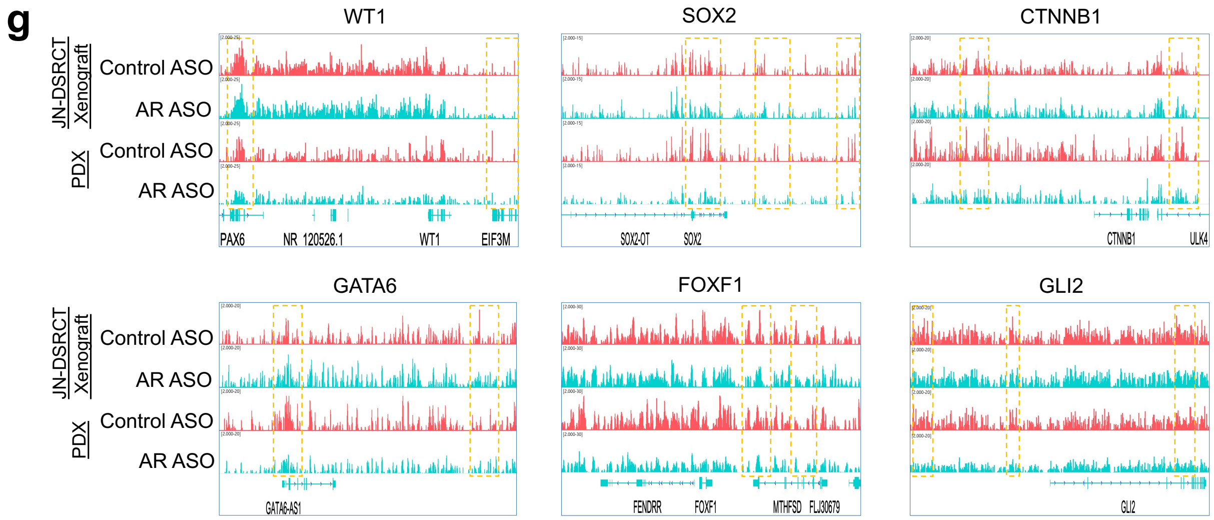

Supplemental Figure 9. AR binding in DSRCT Xenograft and PDX tumors. (a-b) Heatmaps (left panels) and average intensity curves (right panels) of ChIP-seq reads (RPKM; reads per kilobase of transcript per million mapped reads) for AR binding regions. AR binding sites are shown in a 10-kb window (centered on the middle of the binding site) in control ASO and AR ASO in DSRCT xenograft (**a**) and PDX (**b**). **(c-d)** Venn diagram (left) showing the overlap of all AR peaks between control ASO and AR ASO samples in DSRCT xenograft (**c**) and PDX (**d**) to identify the AR-unique or shared binding sites. Dot plot (right) showing significantly enriched pathways for top 5000 AR specific binding sites in DSRCT xenograft (**c**) and PDX (**d**). Dot size represents gene ratio, and colors represents adjusted *p*-values (Fisher's exact test). **(e)** List of top 5 enriched transcription factor (TF) motifs in AR specific binding sites in DSRCT xenograft and PDX. Motifs are identified using HOMER. **(f)** Venn diagram showing the overlap of annotated genes for AR specific gained enhancer peaks in DSRCT xenograft (left) and PDX (right) and upregulated gene list for DSRCT tumors vs. other sarcoma tumors. **(g)** IGV images showing enrichment of AR peaks around WT1, SOX2, CTNNB1, GATA6, FOXF1, and GLI2 genes using aggregate ChIP-seq profiles of Control ASO and AR-ASO samples in DSRCT xenograft (top) and PDX (bottom).

Supplemental Figure 10

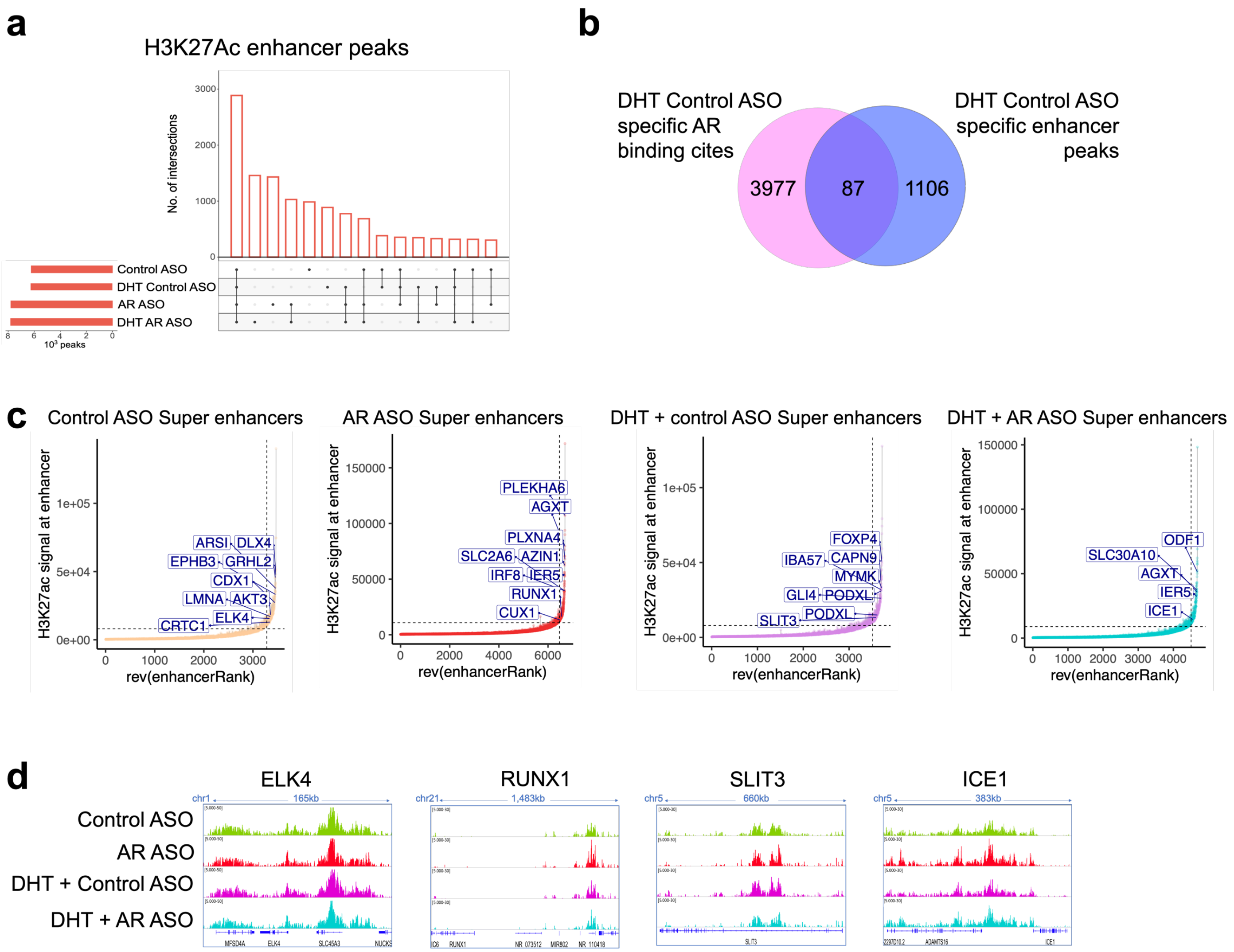

Supplemental Figure 10. Enhancer reprogramming by AR in JN-DSRCT cells. (a) Upset plots displaying unique and shared enhancer regions between Control ASO, AR ASO, DHT + Control ASO, and DHT + AR ASO samples. **(b)** Venn diagram showing the overlap of AR specific enhancer peaks and AR peaks. **(c)** Inflection plot indicating super-enhancers (SEs) identified in Control ASO, AR ASO, DHT + Control ASO, and DHT + AR ASO samples. **(d)** IGV images showing enrichment of super-enhancer H3K27Ac peaks around ELK4, RUNX1, SLIT3, and ICE1 genes using aggregate ChIP-seq profiles of Control ASO, AR ASO, DHT + Control ASO and DHT + AR ASO samples.

Supplemental Figure 11

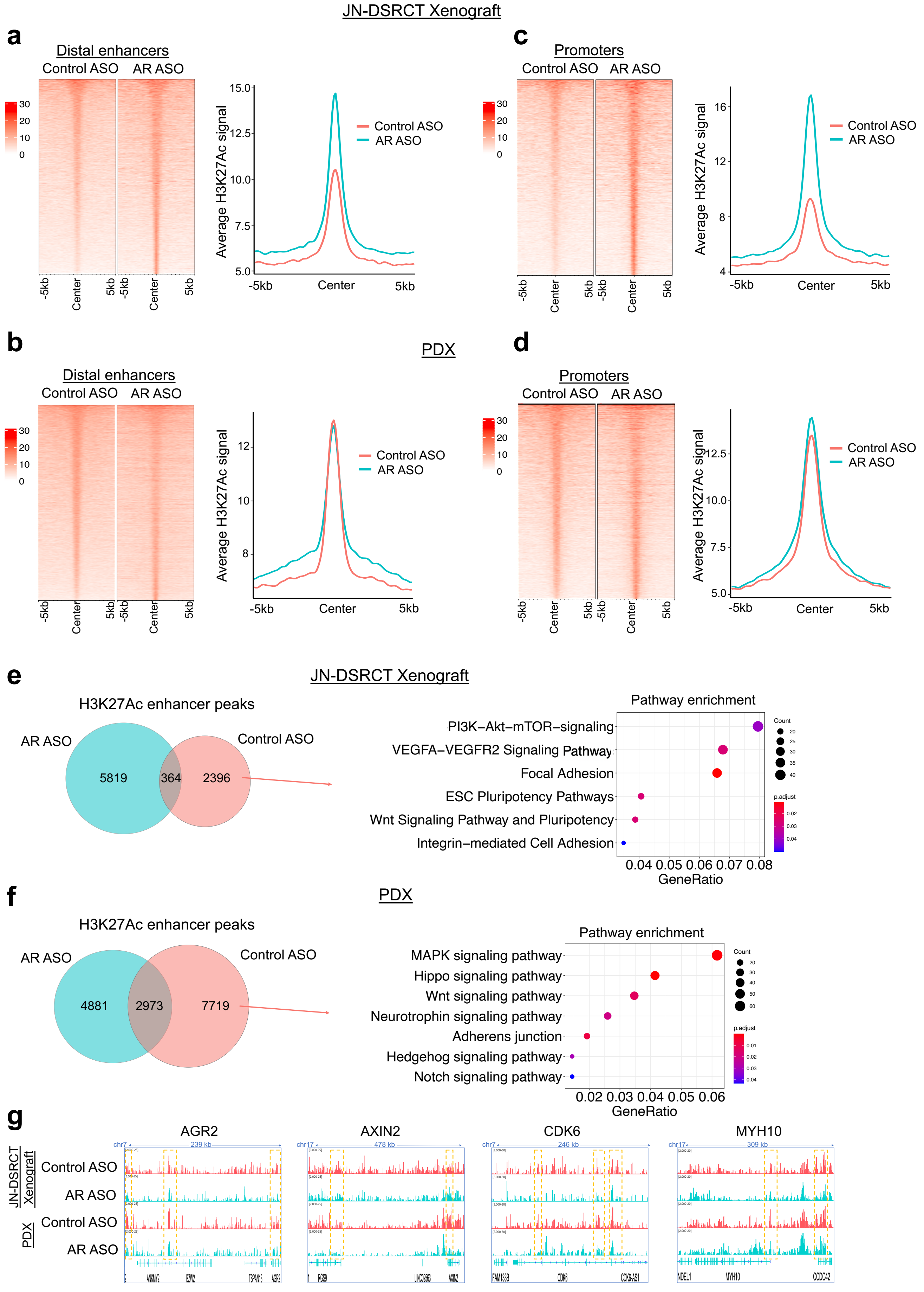

Supplemental Figure 11. Enhancer reprogramming by AR in DSRCT Xenograft and PDX tumors. (a-b) Heatmaps (left panels) and average intensity curves (right panels) of ChIP-seq reads (RPKM; reads per kilobase of transcript per million mapped reads) for distal enhancer regions. H3K27Ac binding sites are shown in a 10-kb window (centered on the middle of the binding site) in Control ASO and AR ASO in DSRCT xenograft (**a**) and PDX (**b**). **(c-d)** Heatmaps (left panels) and average intensity curves (right panels) of ChIP-seq reads (RPKM; reads per kilobase of transcript per million mapped reads) for promoter regions. H3K27Ac binding sites are shown in a 10-kb window (centered on the middle of the binding site) in Control ASO and AR ASO in DSRCT xenograft (**c**) and PDX (**d**). **(e-f)** Venn diagram (left) showing the overlap of all enhancer peaks between control ASO and AR ASO samples in DSRCT xenograft (**e**) and PDX (**f**) to identify the H3K27Ac-unique or shared binding sites. Dot plot (right) showing significantly enriched pathways for top 5000 H3K27Ac specific binding sites in DSRCT xenograft (**e**) and PDX (**f**). Dot size represents gene ratio, and colors represents adjusted *p*-values (Fisher's exact test). **(g)** IGV images showing enrichment of H3K27Ac peaks around AGR2, AXIN2, CDK6, and MYH10 genes using aggregate ChIP-seq profiles of Control ASO and AR-ASO samples in DSRCT xenograft (top) and PDX (bottom).

Supplemental Figure 12

a

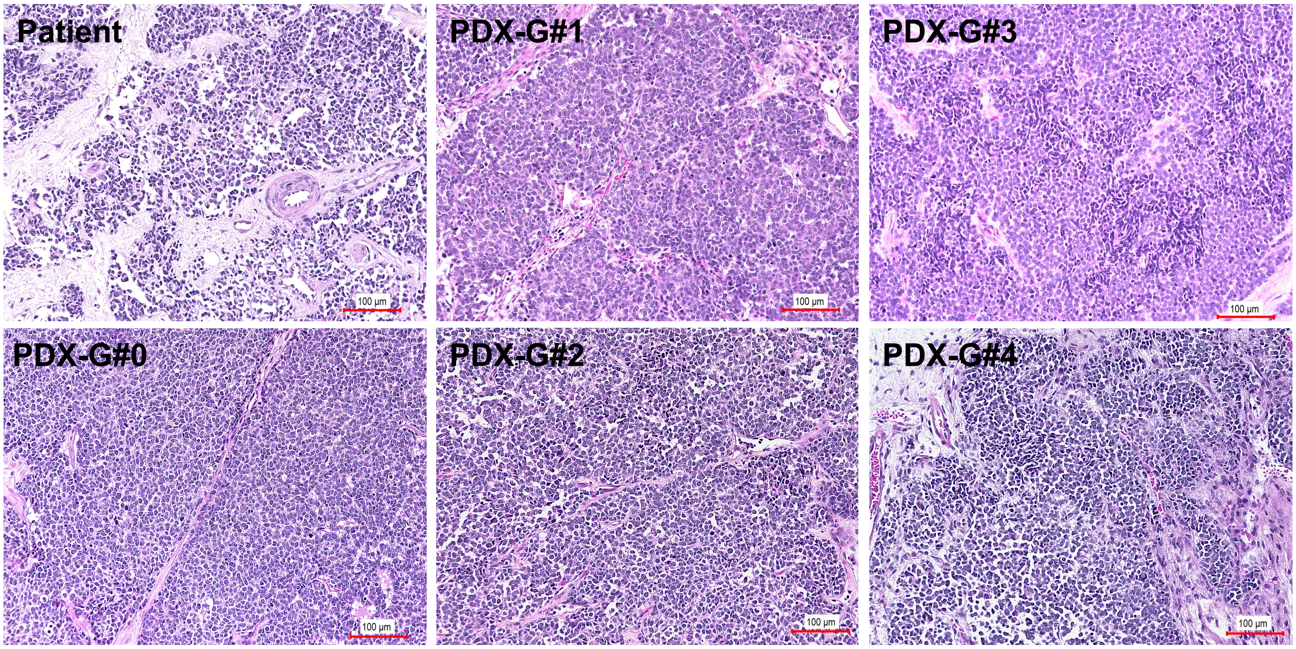

b

	AMEL	CSF1PO	D13S317	D16S539	D18S51	D21S11	D3S1358	D5S818	D7S820	D8S1179	FGA	TH01	TPOX	vWA
**Patient**	X,Y	10,12	8	10,13	12,14	30	14	13	9	11,12	25,26	6,7	8,9	17
**PDX-G0**	X,Y	10,12	8	10,13	12,14	30	14	13	9	11,12	25,26	7	8,9	17
**PDX-G1**	X,Y	10,12	8	10,13	12,14	30	14	13	9	11,12	25,26	7	8,9	17
**PDX-G2**	X,Y	10,12	8	10,13	12,14	30	14	13	9	11,12	25,26	7	8,9	17
**PDX-G3**	X,Y	10,12	8	10,13	12,14	30	14	13	9	11,12	25,26	7	8,9	17
**PDX-G4**	X,Y	10,12	8	10,13	12,14	30	14	13	9	11,12	25,26	7	8,9	17

c

Sample_ID	Gender	DOB	Diagnosis Date	Age @ Diagnosis	Race	Pathology Type	Pathology Grade	Metastatic Disease @ Diagnosis	Metastatic Site	Treatment Modalities	Vital Status
**AHJ-T15**	**Male**	**1/1/04**	**9/2/13**	**9.7**	**White**	**Metastatic**	**High**	**Yes**	**Lung**	**Chemotherapy Surgery**	**Unknown**

Supplemental Figure 12. Histologic and genetic analyses of DSRCT patient and PDX tumors. (a) The DSRCT patient and his 4th-generation PDX demonstrated similar histological features by H&E staining. **(b)** STR profiling of the PDX matched the patient. **(c)** Clinicopathological characteristics of the human tumor used for DSRCT PDX generation.
