# Supplementary material for: The androgen receptor is a therapeutic target in desmoplastic small round cell sarcoma
Source: Nat Commun. 2022 Jun 1;13:3057. doi: 10.1038/s41467-022-30710-z (PMC9160255; doi:10.1038/s41467-022-30710-z)
Supplement: Supplementary file 3 — Description of Additional Supplementary Files [file 41467_2022_30710_MOESM3_ESM.pdf]

Description of Additional Supplementary Files

File Name: Supplementary Data 1
Description: AR specific binding sites and motif enrichment in JN-DSRCT cells.

File Name: Supplementary Data 2
Description: AR-dependent enhancer reprogramming in JN-DSRCT cells.

File Name: Supplementary Data 3
Description: Super enhancers and regulated genes in JN-DSRCT cells.

File Name: Supplementary Data 4
Description: Normalized RPPA data between DSRCT and paired adjacent normal-appearing mesenteric tissues from the same patients.
