# Supplementary material for: The androgen receptor is a therapeutic target in desmoplastic small round cell sarcoma
Source: Nat Commun. 2022 Jun 1;13:3057. doi: 10.1038/s41467-022-30710-z (PMC9160255; doi:10.1038/s41467-022-30710-z)
Supplement: Supplementary file 8 — Reporting Summary [file 41467_2022_30710_MOESM8_ESM.pdf]

nature portfolio

Corresponding author(s): NCOMMS-20-40738B

Last updated by author(s): Mar 29, 2022

Reporting Summary

Nature Portfolio wishes to improve the reproducibility of the work that we publish. This form provides structure for consistency and transparency in reporting. For further information on Nature Portfolio policies, see our Editorial Policies and the Editorial Policy Checklist.

Statistics

For all statistical analyses, confirm that the following items are present in the figure legend, table legend, main text, or Methods section.

n/a	Confirmed	
☐	☒	The exact sample size (*n*) for each experimental group/condition, given as a discrete number and unit of measurement
☐	☒	A statement on whether measurements were taken from distinct samples or whether the same sample was measured repeatedly
☐	☒	The statistical test(s) used AND whether they are one- or two-sided<br>*Only common tests should be described solely by name; describe more complex techniques in the Methods section.*
☐	☒	A description of all covariates tested
☐	☒	A description of any assumptions or corrections, such as tests of normality and adjustment for multiple comparisons
☐	☒	A full description of the statistical parameters including central tendency (e.g. means) or other basic estimates (e.g. regression coefficient) AND variation (e.g. standard deviation) or associated estimates of uncertainty (e.g. confidence intervals)
☐	☒	For null hypothesis testing, the test statistic (e.g. *F*, *t*, *r*) with confidence intervals, effect sizes, degrees of freedom and *P* value noted<br>*Give P values as exact values whenever suitable.*
☐	☒	For Bayesian analysis, information on the choice of priors and Markov chain Monte Carlo settings
☐	☒	For hierarchical and complex designs, identification of the appropriate level for tests and full reporting of outcomes
☐	☒	Estimates of effect sizes (e.g. Cohen's *d*, Pearson's *r*), indicating how they were calculated

Our web collection on statistics for biologists contains articles on many of the points above.

Software and code

Policy information about availability of computer code

Data collection	22Rv1 AR ChIP-seq peaks bed file were downloaded from the Gene Expression Omnibus repository under GSE96652 series.
Data analysis	ChIP-seq raw reads were mapped by bowtie1 to hg19. RPKM normalized bigwigs were generated by deeptools and tracks were visualized with IGV. Peaks were called using macs1.4. Heatmaps were generated using R package EnrichedHeatmap. ChIP-seq peaks were annotated with the nearest genes using ChIPseeker. Super-enhancers were identified using ROSE based on H3K27ac ChIP-seq data.

For manuscripts utilizing custom algorithms or software that are central to the research but not yet described in published literature, software must be made available to editors and reviewers. We strongly encourage code deposition in a community repository (e.g. GitHub). See the Nature Portfolio guidelines for submitting code & software for further information.

Data

Policy information about availability of data

All manuscripts must include a data availability statement. This statement should provide the following information, where applicable:

- Accession codes, unique identifiers, or web links for publicly available datasets
- A description of any restrictions on data availability
- For clinical datasets or third party data, please ensure that the statement adheres to our policy

All ChIP-seq data are available at GEO accession number GSE151380. RNA-seq data are made publicly available in the European Genome-Phenome Archive: DSRCT at EGAS00001004575; liposarcoma at EGAS00001002807; osteosarcoma at EGAS00001003247; chondrosarcoma at EGAS00001004585; and prostate cancer through Subudhi et al.

Field-specific reporting

Please select the one below that is the best fit for your research. If you are not sure, read the appropriate sections before making your selection.

☒ Life sciences ☐ Behavioural & social sciences ☐ Ecological, evolutionary & environmental sciences

For a reference copy of the document with all sections, see nature.com/documents/nr-reporting-summary-flat.pdf

Life sciences study design

All studies must disclose on these points even when the disclosure is negative.

Sample size	The sample size in all our experiments was determined based on the a priori expected experimental variation and statistical power calculations.
Data exclusions	No data were excluded from the analyses.
Replication	All the in vitro and in vivo experiments were at least performed twice, with at least three replicates for in vitro cell proliferation assays and >5 mice per group for in vivo studies, to confirm the reproducibility of our results.
Randomization	The animals were randomly assigned to treatment groups once tumors reached sufficient size to begin drug treatment. Care was taken to ensure all animals appeared healthy before enrollment into a drug treatment group.
Blinding	RPPA measurements were performed by blinded 3rd party labs and tissue microarray (TMA) protein expression scoring was automated using a computer-based algorithm.

Reporting for specific materials, systems and methods

We require information from authors about some types of materials, experimental systems and methods used in many studies. Here, indicate whether each material, system or method listed is relevant to your study. If you are not sure if a list item applies to your research, read the appropriate section before selecting a response.

Materials & experimental systems

n/a	Involved in the study	
☐	☒	Antibodies
☐	☒	Eukaryotic cell lines
☒	☐	Palaeontology and archaeology
☐	☒	Animals and other organisms
☐	☒	Human research participants
☒	☐	Clinical data
☒	☐	Dual use research of concern

Methods

n/a	Involved in the study	
☐	☒	ChIP-seq
☒	☐	Flow cytometry
☒	☐	MRI-based neuroimaging

Antibodies

Antibodies used	Please see provided "Supplemental materials, methods" document.
Validation	Antibody information and protocols used in RPPA is provided in the following link: https://www.mdanderson.org/research/researchresources/core-facilities/functional-proteomics-rppa-core/antibody-information-and-protocols.html

Eukaryotic cell lines

Policy information about cell lines

Cell line source(s)	JN-DSRCT cell line exhibiting a pathognomonic t(11;22)(p13;q12) translocation was generously provided from Dr. M Kikuchi's laboratory (Fukuoka University, Fukuoka, Japan). LnCaP and TC71 cell lines are provided by MDA cell lines core facility.
Authentication	All available cell lines in Dr. Ludwig's lab are registered within the MDA characterized cell line core (CCLC). Each cell line identity was validated twice per year in MDA CCLC using short-tandem repeat (STR) fingerprinting with an AmpFLSTR Identifier kit.
Mycoplasma contamination	All our cell lines were tested twice per year for mycoplasma contamination using the MycoAlert Detection Kit (Lonza Group Ltd.) according to the manufacturer's protocol. Additionally, cells lines are sent for 3rd-party mycoplasma testing using a sensitive PCR testing approach any time a collection of cells are cryopreserved.

Commonly misidentified lines (See ICLAC register)	Our CCLC misidentified cell line list is available at: https://www.mdanderson.org/content/dam/mdanderson/documents/core-facilities/Characterized%20Cell%20Line%20Core%20Facility/Misidentified%20cell%20lines_CCLC.pdf

Animals and other organisms

Policy information about studies involving animals; ARRIVE guidelines recommended for reporting animal research

Laboratory animals	Male adult (6 week old) NOD (SCID)-IL-2Rgnull mice (The Jackson Laboratory; Farmington, CT) were subcutaneously injected with JN-DSRCT cells (5X106 cells/animal) or received PDX explants (2 mm) to generate xenografts.
Wild animals	The study did not involve wild animals.
Field-collected samples	All mice were maintained under barrier conditions and treated using protocols approved by The University of Texas MD Anderson Cancer Center's Institutional Animal Care and Use Committee. Once their tumors reached a volume of 150 mm3, 5 mice per group received enzalutamide (25 mg/kg IP daily, 5 times per week), or AR ASOs (25 or 50 mg/kg subcutaneously daily, 5 times per week), or control ASOs (50 mg/kg subcutaneously daily, 5 times per week), or a placebo control (sterile vehicle buffer). Tumor volumes were measured using digital calipers at study initiation and 2–5 times per week after that for up to 85 days, or until their tumors reached 1500 mm3, whichever came first. A Kaplan-Meier analysis was performed to assess drug efficacy.
Ethics oversight	All experiments were conducted per protocols and conditions approved by the University of Texas MD Anderson Cancer Center (MDACC; Houston, TX) Institutional Animal Care and Use Committee (eACUF Protocols #00000712-RN03).

Note that full information on the approval of the study protocol must also be provided in the manuscript.

Human research participants

Policy information about studies involving human research participants

Population characteristics	The charts and electronic medical records of patients with a confirmed diagnosis of DSRCT were included for analysis. We identified 60 DSRCT patients treated at MDACC from 1990 to 2019 to generate a TMA.
Recruitment	Specialist pathologists used clinical information, immunohistochemistry, and cytogenic analysis for the EWSR1-WT1 fusion to confirm the DSRCT diagnoses.
Ethics oversight	The collection of DSRCT tumor patients was approved by the Institutional Review Board of MDACC under the LAB08-0151 and LAB04-0890 protocols and conducted in compliance with the principals of the Declaration of Helsinki.

Note that full information on the approval of the study protocol must also be provided in the manuscript.

ChIP-seq

Data deposition

☒ Confirm that both raw and final processed data have been deposited in a public database such as GEO.

☒ Confirm that you have deposited or provided access to graph files (e.g. BED files) for the called peaks.

Data access links
May remain private before publication.

https://www.ncbi.nlm.nih.gov/geo/query/acc.cgi?acc=GSE151380 ; with reviewer token: qpazsmqgdzyhrwh

Files in database submission

Accession Title Release date Status Supplementary files

GSE151380 Genome-wide AR and H3K27Ac binding profiles in DSRCT cells, xenograft and PDX mouse tumors 9/1/22
GSM4577068 Control ASO AR_ChIPseq Sep 01, 2022 approved BW BED
GSM4577069 Control ASO H3K27ac_ChIPseq Sep 01, 2022 approved BW BED
GSM4577070 Control ASO Input DNA Sep 01, 2022 approved BW
GSM4577071 AR ASO AR_ChIPseq Sep 01, 2022 approved BW BED
GSM4577072 AR ASO H3K27ac_ChIPseq Sep 01, 2022 approved BW BED
GSM4577073 AR ASO Input DNA Sep 01, 2022 approved BW
GSM4577074 DHT Control ASO AR_ChIPseq Sep 01, 2022 approved BW BED
GSM4577075 DHT Control ASO H3K27ac_ChIPseq Sep 01, 2022 approved BW BED
GSM4577076 DHT Control ASO Input DNA Sep 01, 2022 approved BW
GSM4577077 DHT AR ASO AR_ChIPseq Sep 01, 2022 approved BW BED
GSM4577078 DHT AR ASO H3K27ac_ChIPseq Sep 01, 2022 approved BW BED
GSM4577079 DHT AR ASO Input DNA Sep 01, 2022 approved BW
GSM5689227 JN-DSRCT_Control-ASO AR_ChIPseq Sep 01, 2022 approved BW BED
GSM5689228 JN-DSRCT_Control-ASO H3K27ac_ChIPseq Sep 01, 2022 approved BW BED
GSM5689229 JN-DSRCT_Control-ASO Input DNA Sep 01, 2022 approved BW
GSM5689230 JN-DSRCT_AR AR_ChIPseq Sep 01, 2022 approved BW BED
GSM5689231 JN-DSRCT_AR H3K27ac_ChIPseq Sep 01, 2022 approved BW BED
GSM5689232 JN-DSRCT_AR Input DNA Sep 01, 2022 approved BW
GSM5689233 DSRCT-PDX1_Control_ASO AR_ChIPseq Sep 01, 2022 approved BW BED
GSM5689234 DSRCT-PDX1_Control_ASO H3K27ac_ChIPseq Sep 01, 2022 approved BW BED

GSM5689235 DSRCT-PDX1_Control_ASO Input DNA Sep 01, 2022 approved BW
GSM5689236 DSRCT-PDX1_AR_ASO AR_ChIPseq Sep 01, 2022 approved BW BED
GSM5689237 DSRCT-PDX1_AR_ASO H3K27ac_ChIPseq Sep 01, 2022 approved BW BED
GSM5689238 DSRCT-PDX1_AR_ASO Input DNA Sep 01, 2022 approved BW
GEO (GSE108687) RPPA (Fig. 1A-B)
GEO (GSE178406) RPPA, Figure 6A
GEO (GSE108687) RPPA, Supplemental (Fig. 1A-B)
GEO (GSE178406) RPPA, Supplemental Figure 6F-G
EGAS00001004575 RNA-Seq for DSRCT (Fig 2E&F)
EGAS00001002807 RNA-Seq for Liposarcoma (Fig 2E&F)
EGAS00001003247 RNA-Seq for Osteosarcoma (Fig 2E&F)
EGAS00001004585 RNA-Seq for Chondrosarcoma (Fig 2E&F)
EGAS00001004050 RNA-Seq for Prostate Cancer (Fig 2E&F)

Genome browser session (e.g. UCSC): N/A

Methodology

Replicates: One replicate was performed

Sequencing depth: 36bp single end reads were aligned to the hg19 genome. At least 25 million uniquely mapped reads were used for H3K27Ac mark, At least 10 million uniquely mapped reads were used for AR ChIP-seq in cell lines. At least 20 million uniquely mapped reads were used for both H3K27Ac and AR ChIP-seq in mouse tumor samples.

Antibodies: H3K27ac (Abcam ab4729) & AR (CST #5153)

Peak calling parameters: Peaks were called using MACS14; For final analysis, p-value cutoff 1e-9 used for H3K27ac and 1e-7 used for AR in cell lines; p-value cutoff 1e-5 used for H3K27ac and AR in mouse tumors.
#macs peak calling
macs14 -t -c --keep-dup all -f BAM -g -p 1e-5 -n

Data quality: For H3K27ac, all of the samples have more than 15000 peaks above a 5-fold cut-off and FDR less than 5%. For AR, all of the samples have more than 8000 peaks above a 5-fold cut-off and FDR less than 5%.

Software:
FastQC (v. 0.11.5) and MultiQC (v. 1.8) was used to determine quality of raw fastq reads.
Bowtie1 (v. 1.2.2) was used for alignment.
Samblaster (v. 0.1.24) was used to remove duplicates.
Sambamba (v. 0.6.6)was used for random sampling.
Samtools (v. 1.2)was used to sort and index.
Macs14 (v. 1.4.2) was used to identify peaks.
Deeptools (v. 3.1.3) was used to generate bigWig files.
IGV (v. 2.3.59) was used to display bigWig files.
R package (v. 4.1.0) EnrichedHeatmap was used to generate Heatmaps.
ChIPseeker (v. 1.30.3)was used to annotate ChIP-seq peaks with the nearest genes.
ROSE (v. 20200511) was used to identify Super-enhancers based on H3K27ac ChIP-seq data.
HOMER (v. 4.10) was used for motif calls.
