# Supplementary material for: The androgen receptor is a therapeutic target in desmoplastic small round cell sarcoma
Source: Nat Commun. 2022 Jun 1;13:3057. doi: 10.1038/s41467-022-30710-z (PMC9160255; doi:10.1038/s41467-022-30710-z)

Original Blot Images From Figure 1C

Primary Ab:
CST#5153
Clone#D6F11

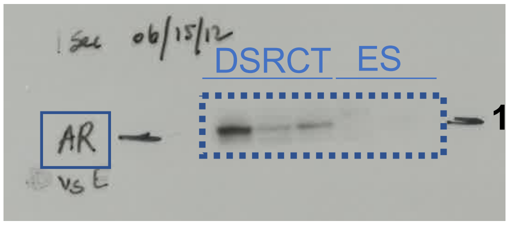

110 kDa

Primary Ab:
CST#13198
Clone#D3Z1E

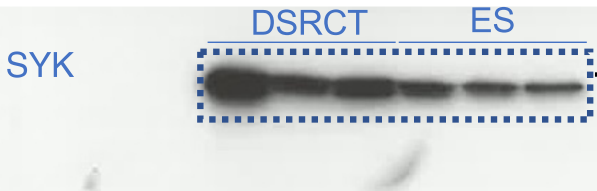

72 kDa

Primary Ab:
CST#46687
Clone#D1M9X

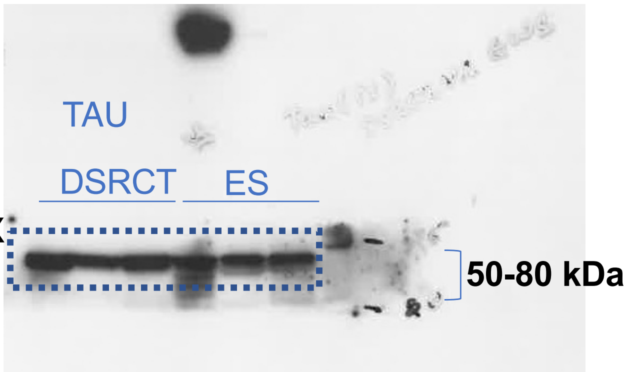

Primary Ab:
CST#37805
Clone#D3W6Y

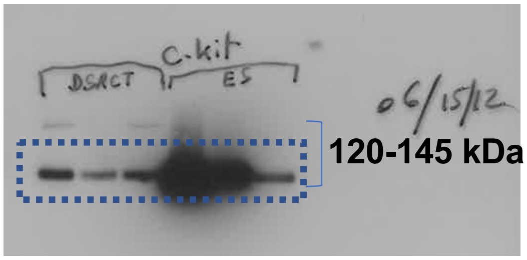

Primary Ab:
CST#4858
Clone#D57.2.2E

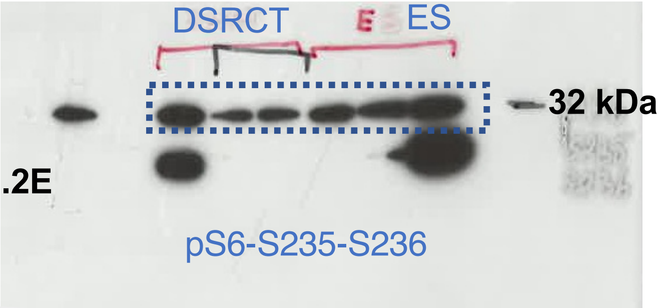

Primary Ab:
CST#3700
Clone#8H10D10

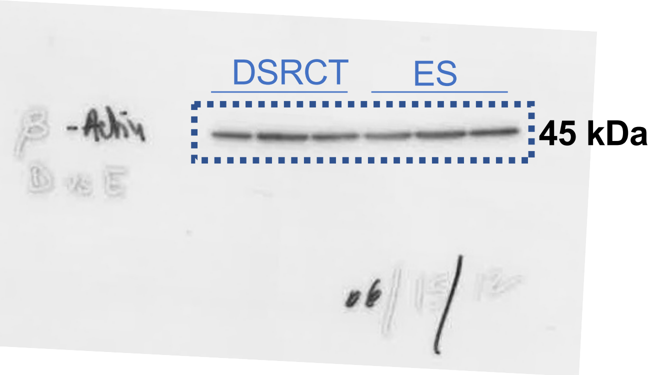

45 kDa

Original Blot Images From Figure 2C

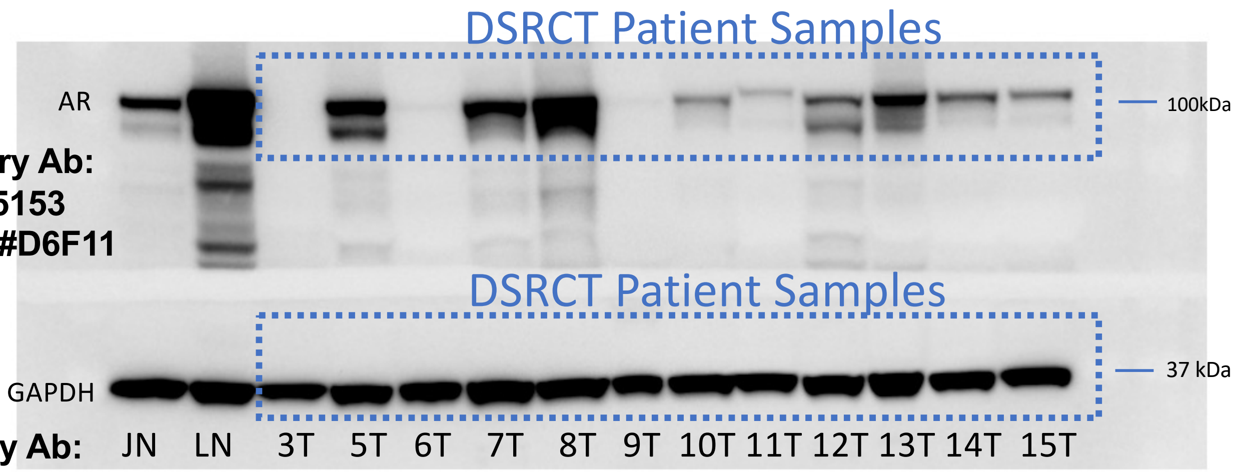

Primary Ab:
CST#5153
Clone#D6F11

Primary Ab:
CST#5174
Clone#D16H11

JN: JN-DSRCT Cell Line
LN: LNCAP Cell Line
DSRCT Patient samples (3T-15T)
Androgen receptor: 110kDa

Original Blot Images From Figure 3B

Primary Ab:
CST#5153
Clone#D6F11

Primary Ab:
CST#5174
Clone#D16H11

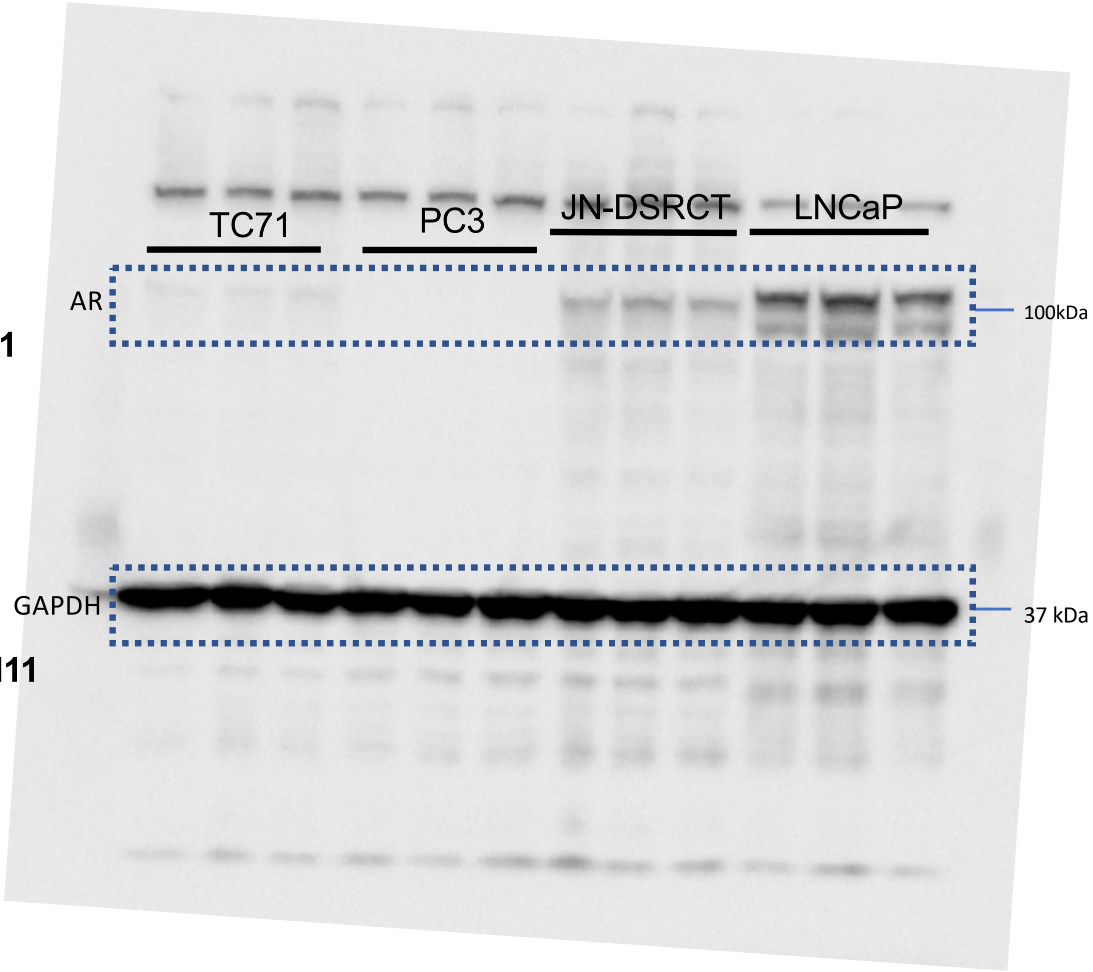

Original Blot Images From Figure 3G

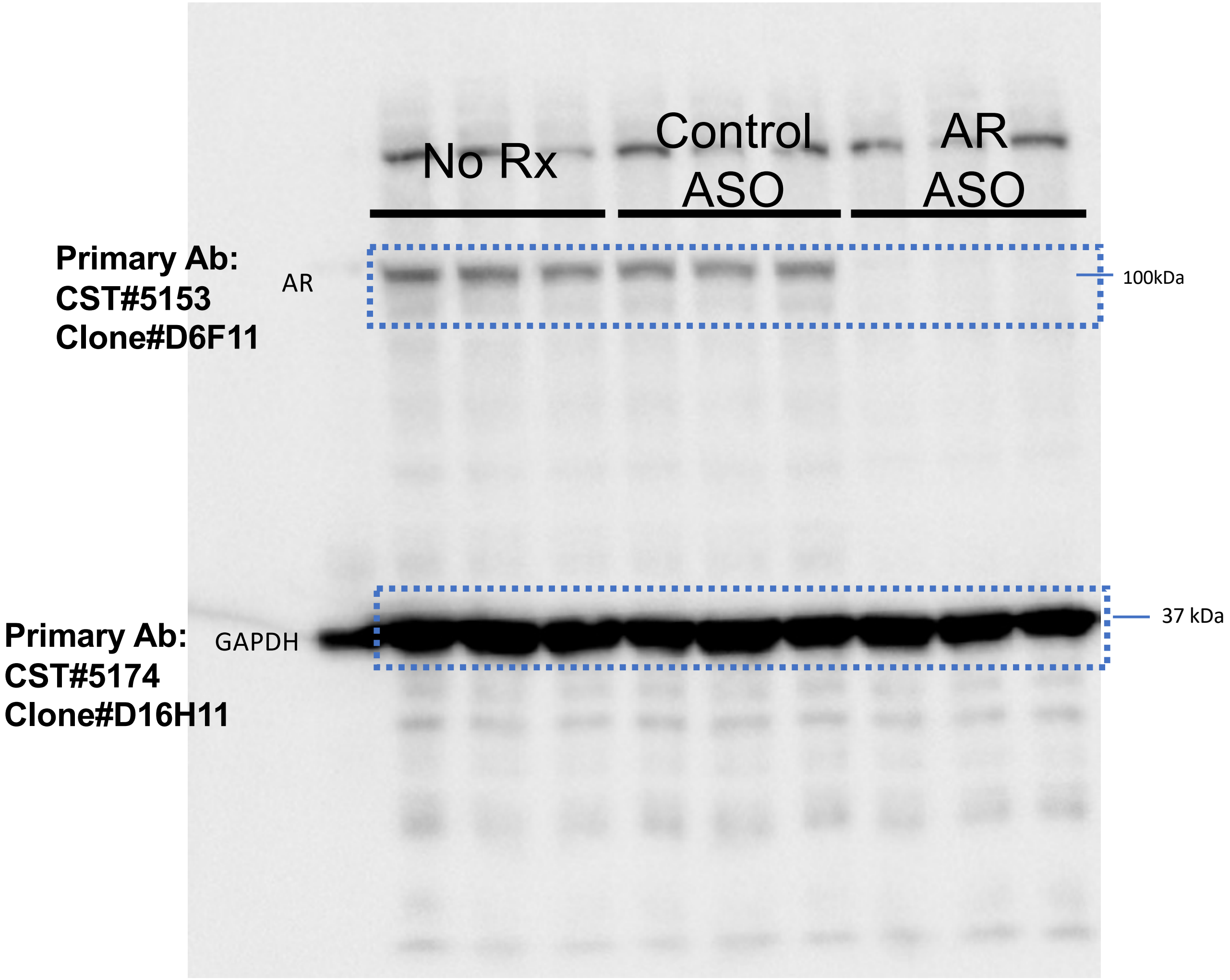

Original Blot Images From Figure 5B

PDX DSRCT

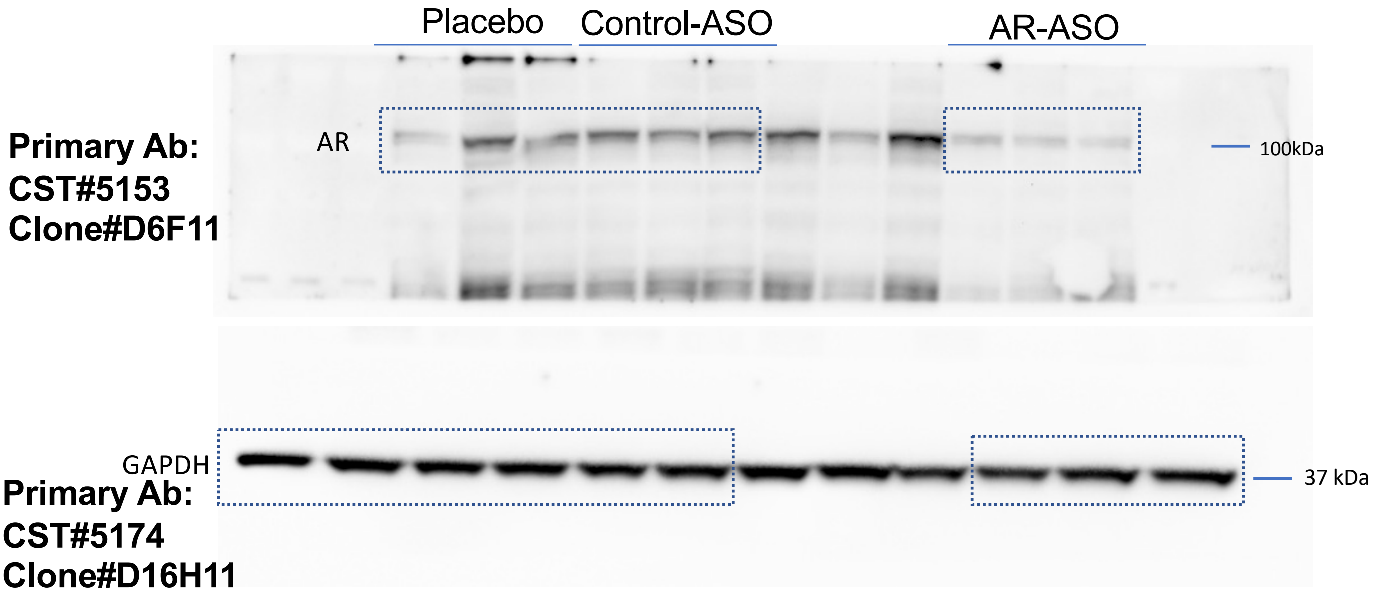

JN-DSRCT Xenograft

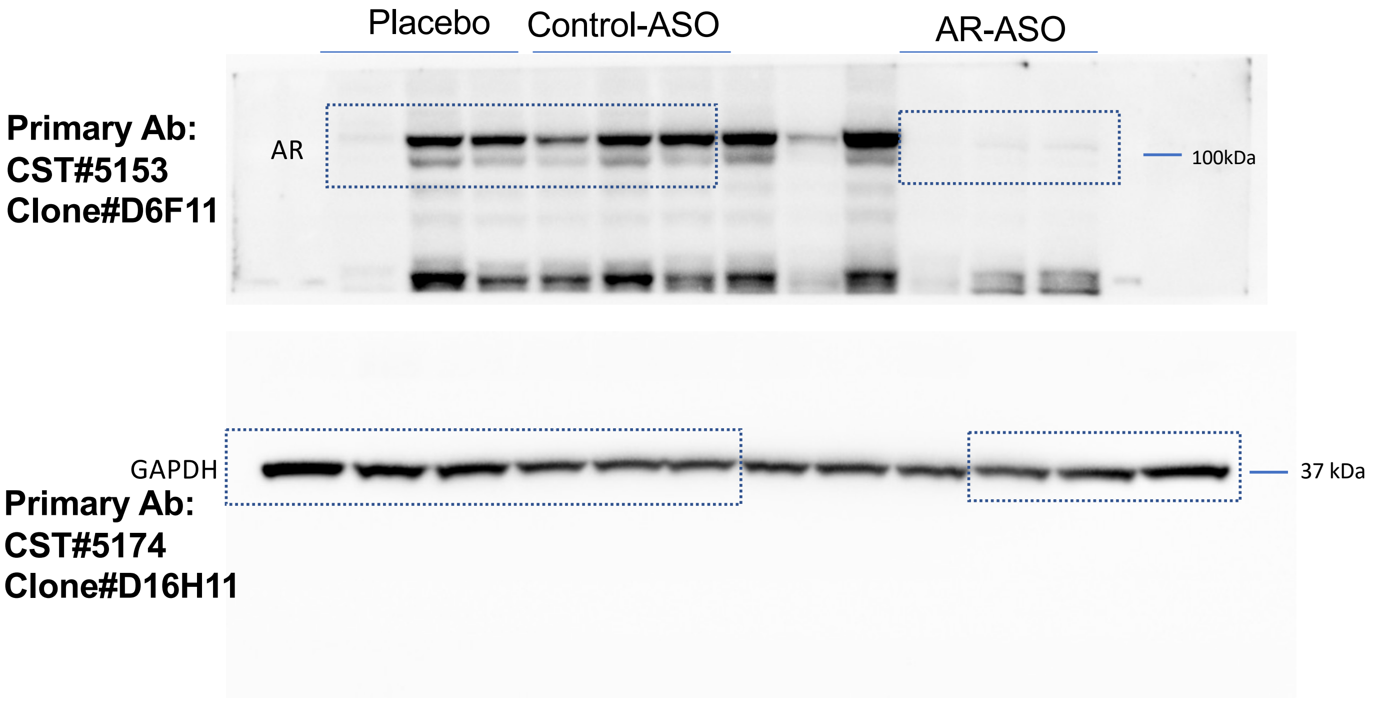

Original Blot Images From Supplemental Figure 1C

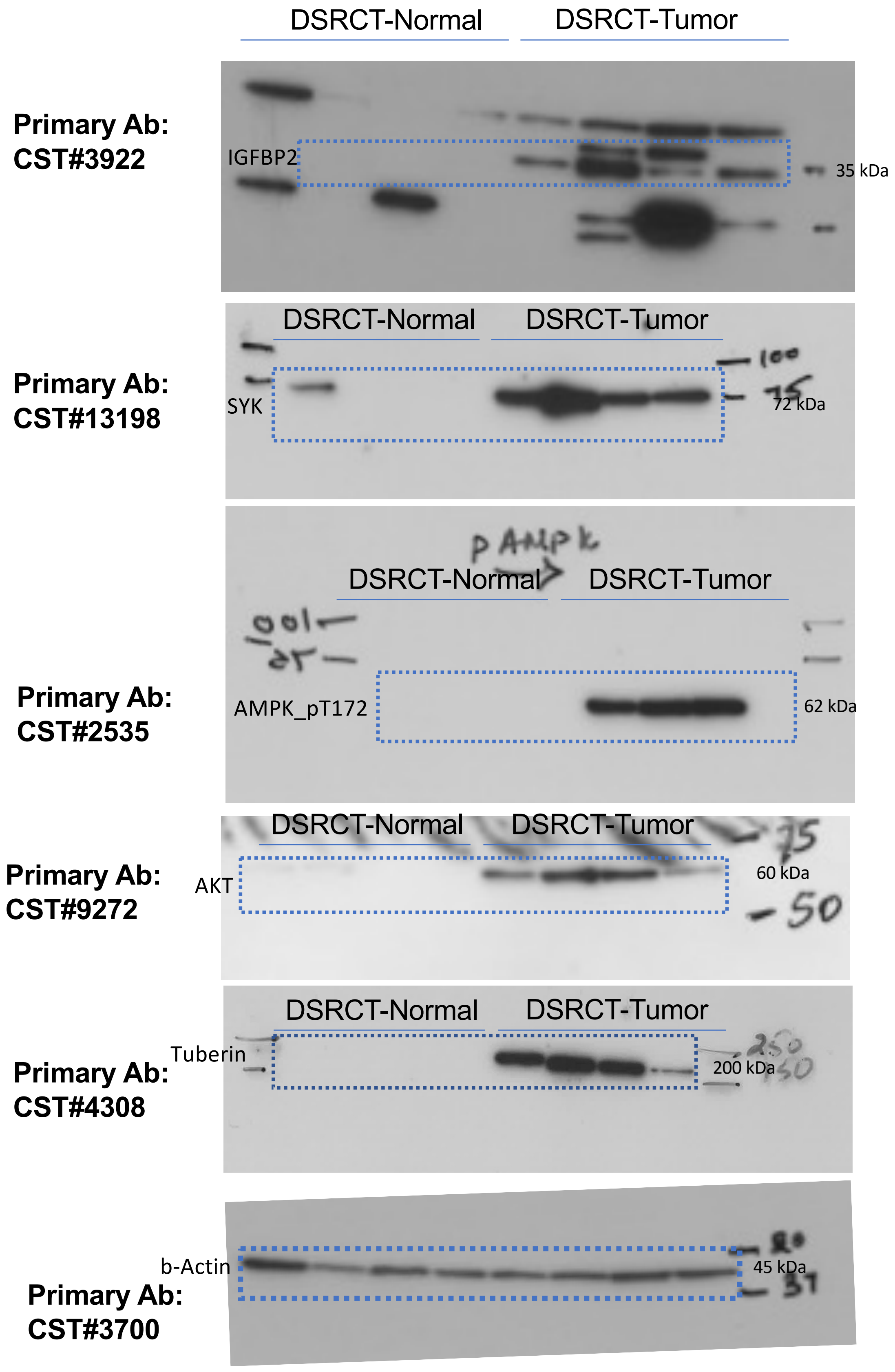

Original Blot Images From Supplemental Figure 3A

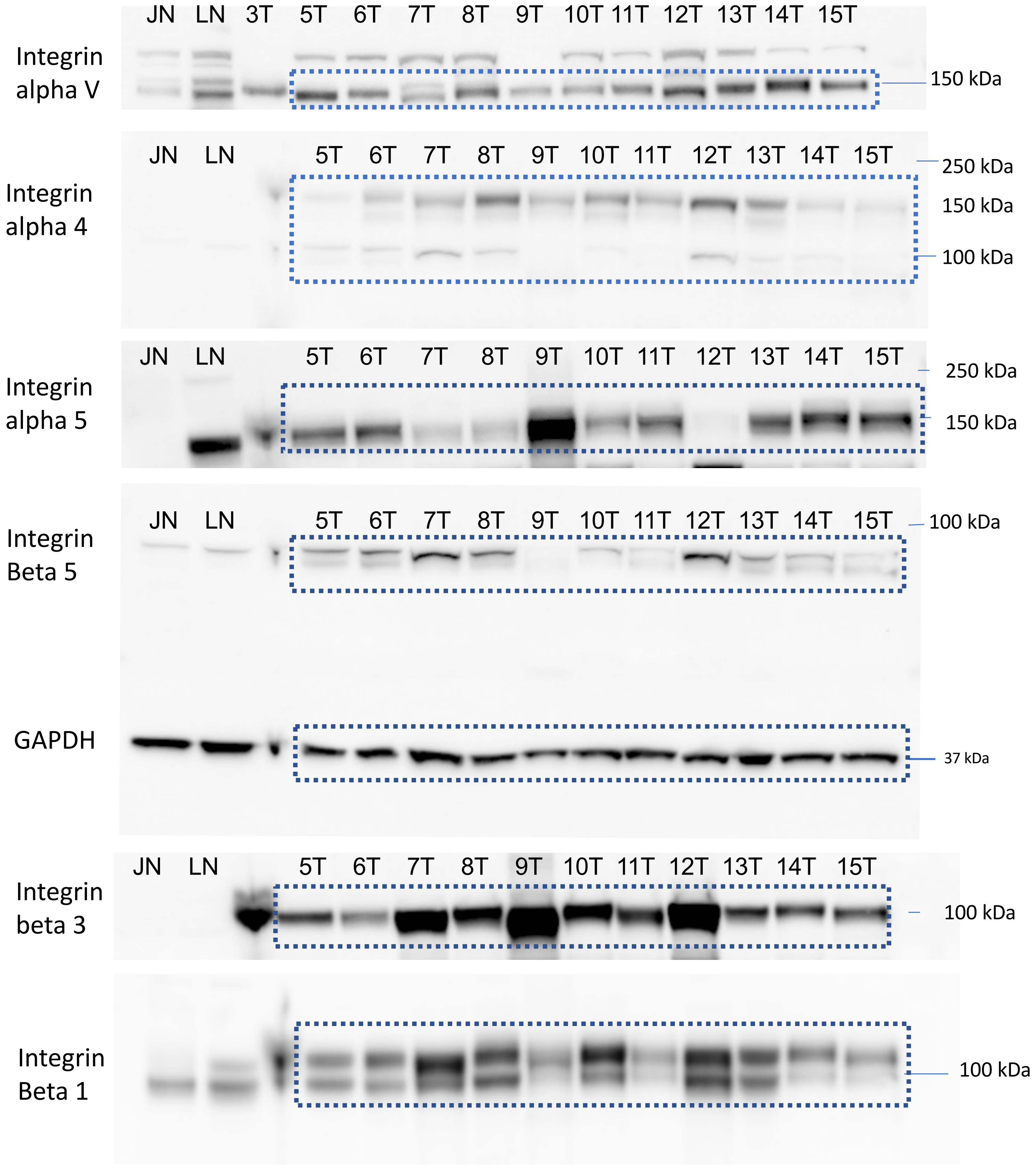

JN: JN-DSRCT
LN: LNCAP

Original Blot Images From Supplemental Figure 3B

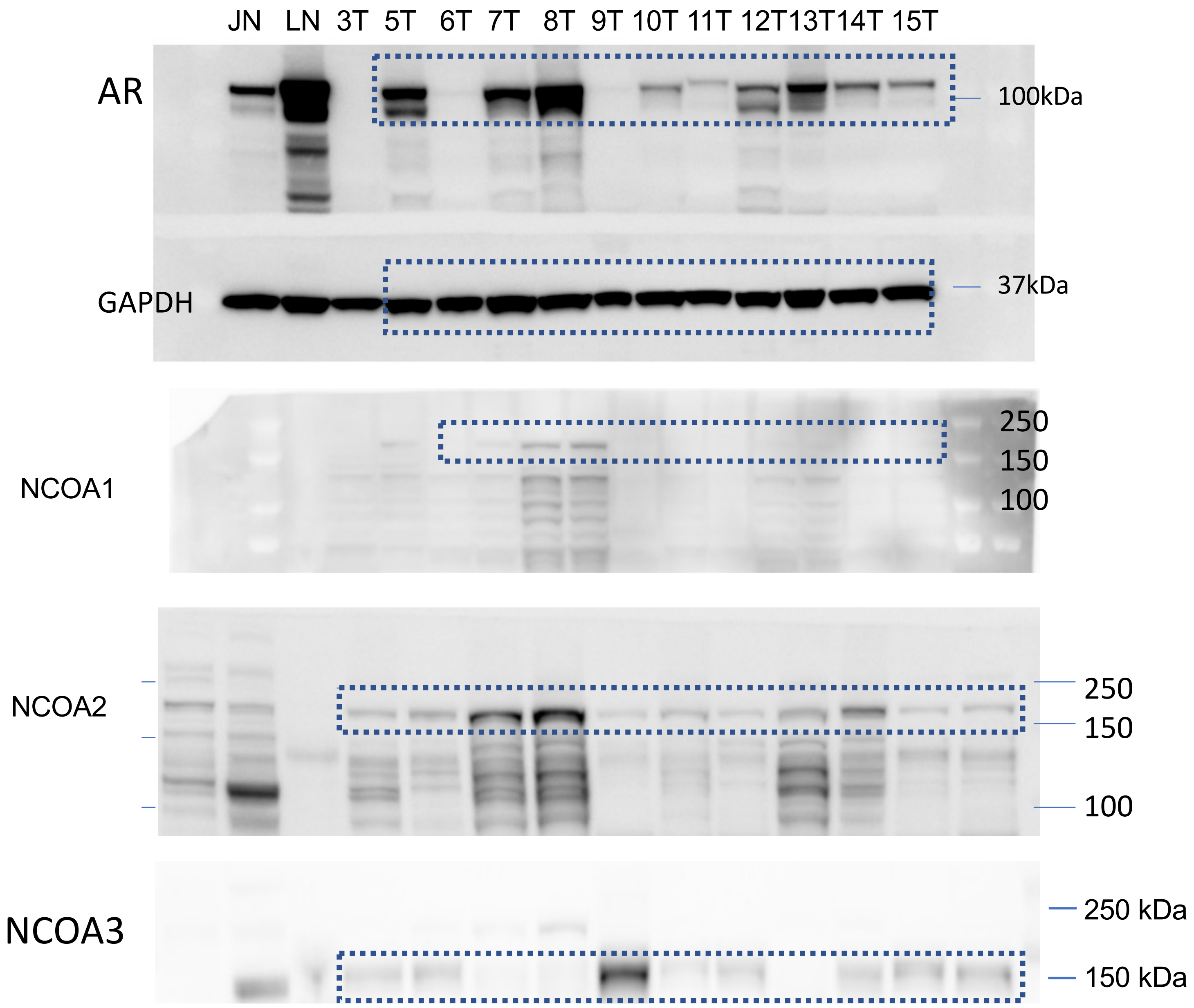

Original Blot Images From Supplemental Figure 3C

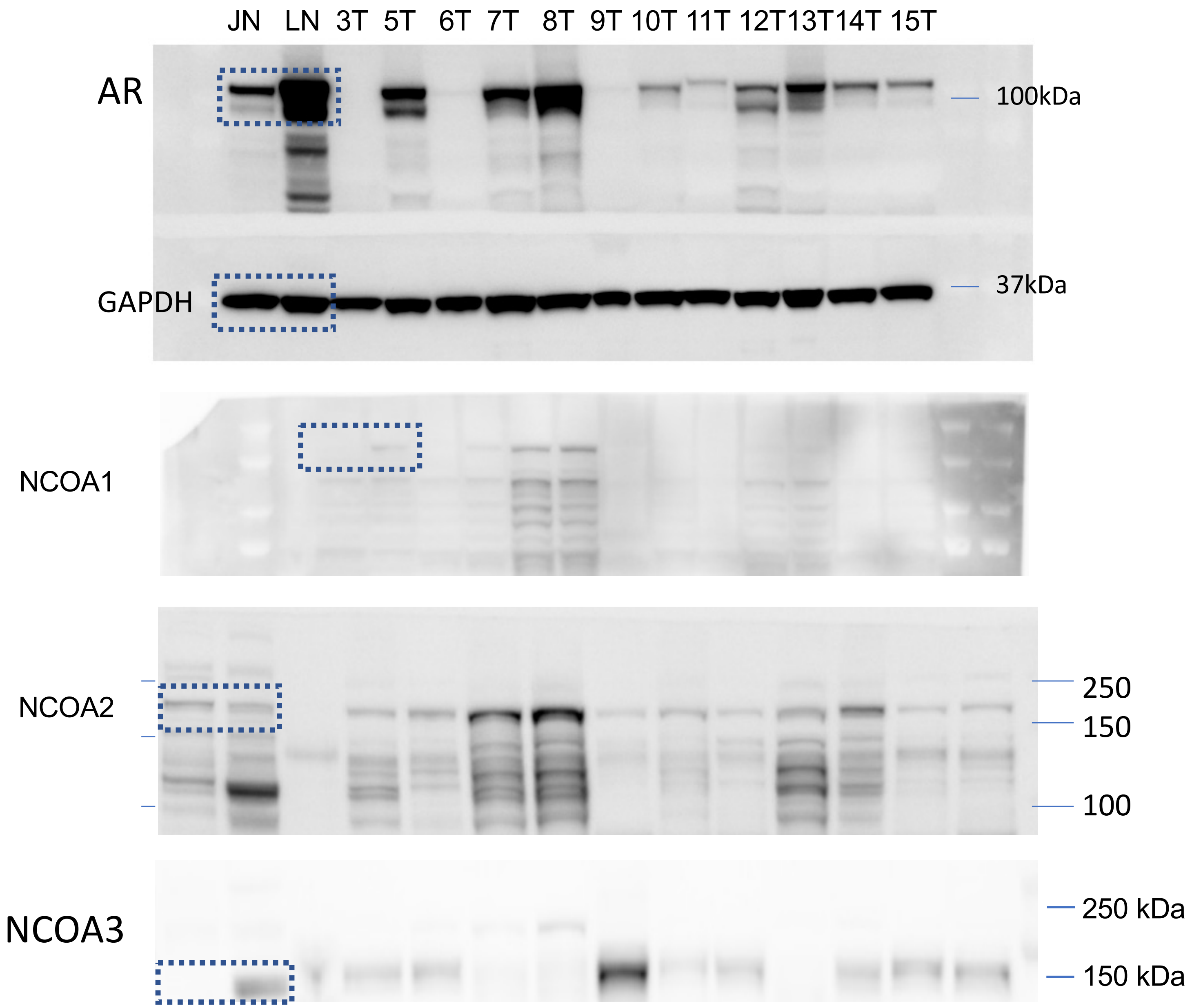

Supplement: Supplementary file 9 — Source Data [file 41467_2022_30710_MOESM9_ESM.zip › Source Data - Original Blot Images.pdf]
